# Cilia-based peptidergic signaling

**DOI:** 10.1371/journal.pbio.3000566

**Published:** 2019-12-06

**Authors:** Raj Luxmi, Dhivya Kumar, Richard E. Mains, Stephen M. King, Betty A. Eipper

**Affiliations:** 1 Department of Neuroscience, University of Connecticut Health Center, Farmington, Connecticut, United States of America; 2 Department of Molecular Biology and Biophysics, University of Connecticut Health Center, Farmington, Connecticut, United States of America; 3 Electron Microscopy Facility, University of Connecticut Health Center, Farmington, Connecticut, United States of America; Rheinische Friedrich-Wilhelms-Universitat Bonn, GERMANY

## Abstract

Peptide-based intercellular communication is a ubiquitous and ancient process that predates evolution of the nervous system. Cilia are essential signaling centers that both receive information from the environment and secrete bioactive extracellular vesicles (ectosomes). However, the nature of these secreted signals and their biological functions remain poorly understood. Here, we report the developmentally regulated release of the peptide amidating enzyme, peptidylglycine α-amidating monooxygenase (PAM), and the presence of peptidergic signaling machinery (including propeptide precursors, subtilisin-like prohormone convertases, amidated products, and receptors) in ciliary ectosomes from the green alga *Chlamydomonas*. One identified amidated PAM product serves as a chemoattractant for mating-type minus gametes but repels plus gametes. Thus, cilia provide a previously unappreciated route for the secretion of amidated signaling peptides. Our study in *Chlamydomonas* and the presence of PAM in mammalian cilia suggest that ciliary ectosome-mediated peptidergic signaling dates to the early eukaryotes and plays key roles in metazoan physiology.

## Abstract 2

Peptide-mediated intercellular communication is an essential process, and those peptides are usually released from secretory granules. This study demonstrates that peptidergic signals are also secreted through vesicular ectosomes that bud from the ciliary membrane.

## Introduction

Our understanding of peptidergic signaling grew out of studies on peptides like vasopressin, whose storage in nerve terminals facilitated its purification [1]; opioid peptides, which were identified based on their ability to interact with specific G protein–coupled receptors (GPCRs) [2]; and insulin, whose loss causes diabetes mellitus [3]. Production of these signaling molecules begins in the endoplasmic reticulum, with synthesis of a prepropeptide. Following signal peptide removal, subtilisin-like proteases cleave the propeptides into smaller products, many of which are subjected to posttranslational modification as they move through the Golgi complex and into secretory granules [3–5]. Secretion of the stored product occurs when triggered by an appropriate stimulus.

Based on extensive studies of peptidergic signaling in diverse metazoan invertebrates such as *Drosophila*, *Caenorhabditis elegans*, sea urchins, and placozoans, it is clear that the proteins involved in peptide synthesis, storage, and regulated release are highly conserved [6–9]. Luminal pH and calcium control activation of the subtilisin-like prohormone convertases, triggering endoproteolytic cleavage at pairs of basic amino acids [3–5]. In many cases, exoproteolytic removal of exposed basic residues reveals a C-terminal glycine. Peptidylglycine α-amidating monooxygenase (PAM) catalyzes the two-step conversion of its peptidylglycine substrates into α-amidated products [10,11]. Amidation is often essential for bioactivity and peptide stability in the extracellular environment [11].

We recently identified PAM in *Chlamydomonas reinhardtii*, a unicellular green alga not known to engage in peptidergic signaling [12]. As in vertebrates, the peptidylglycine α-hydroxylating (PHM) domain of *C*. *reinhardtii* PAM (CrPAM), a Type I integral membrane protein, catalyzes the stereospecific hydroxylation of the α-carbon of glycine; the peptidyl-α-hydroxyglycine α-amidating lyase (PAL) domain cleaves the Cα-N bond, releasing glyoxylate and generating the amidated peptide [12]. Bioinformatic studies identified hundreds of potential prepropeptides encoded by the *C*. *reinhardtii* genome, along with putative subtilisin-like prohormone convertases [13]. However, secretory granules have not been observed in *C*. *reinhardtii*. CrPAM is localized to Golgi and ciliary membranes [12]. Knockdown of CrPAM expression impaired ciliogenesis, preventing extension of the axoneme beyond the transition zone. The ciliary localization of PAM, along with a role in ciliogenesis, are conserved in flatworms, zebrafish, and mice [14,15].

Cilia are ancient microtubule-based organelles that protrude from the cell surface and play key roles in cell movement, fluid flow, and signaling [16]. Acting as antennae, cilia detect environmental signals and transmit that information to the rest of the cell. Key cilia-based signaling pathways include hedgehog and noncanonical Wnt (planar cell polarity), as well as GPCR-mediated signaling by peptides, odorants, and light [17–19]. Studies in *C*. *reinhardtii* and *C*. *elegans* demonstrated that cilia transmit signals in the form of bioactive vesicular ectosomes that bud from the ciliary membrane [20–23]; in *C*. *reinhardtii*, this is the only membrane directly exposed to the environment. Ectosomes released by vegetative *C*. *reinhardtii* cells contain a subtilisin-like lytic enzyme required to release mitotic progeny from the mother cell wall [20]. During sexual reproduction, ectosomes derived from mating gametes contain factors necessary for gamete activation and successful mating [24]. In *C*. *elegans*, ectosomes released by hermaphrodites control male sexual behavior [21].

Here, we demonstrate that CrPAM is secreted in ciliary ectosomes in a developmentally regulated manner during gametogenesis and mating. We find that ectosomes released by mating *C*. *reinhardtii* cells contain multiple propeptides, the enzymes needed to convert these precursors into amidated products, the amidated products themselves, and multiple potential peptide receptors. Furthermore, we show that one ciliary ectosome-associated amidated peptide modulates the chemotactic behavior of *C*. *reinhardtii* gametes. Thus, ciliary ectosomes may function in a cell-autonomous manner to both receive and transmit signals. Considering the ubiquity of peptide-mediated intercellular communication together with the ancient origin of cilia in early eukaryotes, it seems unlikely that the cilia-based peptidergic signaling described here is confined to the chlorophyte algae. Our data predict that peptide secretion through cilia plays a key role throughout metazoan physiology.

## Results

### The *C*. *reinhardtii* genome encodes proteins with the characteristics of preproneuropeptides and the enzymes required for their processing

**Fig 1:**
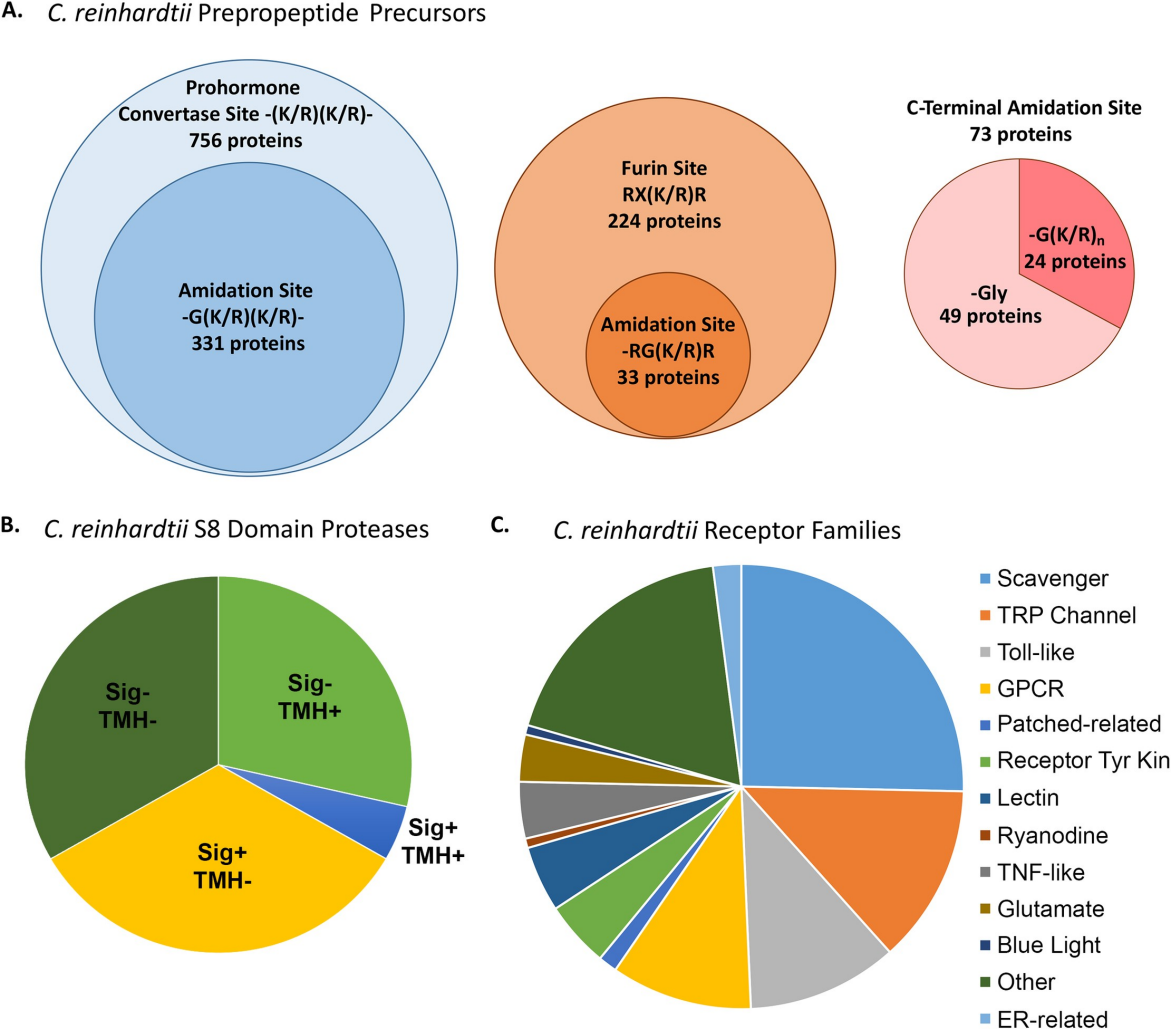
The *C*. *reinhardtii* genome encodes the proteins required for peptidergic signaling. **A.** Potential prepropeptides with predicted prohormone convertase (blue circle) or furin (brown circle) cleavage sites were identified previously [13]; the number that could generate one or more amidated product(s) is indicated by sub-circles. Proteins with potential C-terminal amidation sites were subdivided into those that could be amidated without (pink) or with (red) the participation of a carboxypeptidase B–like enzyme. **B.** The *C*. *reinhardtii* genome encodes 21 subtilisin-like S8 domain-containing proteases that were categorized based on the predicted presence (+) or absence (−) of a signal sequence (Sig) and/or TMH. **C.** A gene annotation screen identified 146 *C*. *reinhardtii* receptors, which were classified into 12 groups on the basis of their putative structure/function. ER, endoplasmic reticulum; GPCR, G protein–coupled receptor; TMH, transmembrane helix; TNF, tumor necrosis factor; TRP, transient receptor potential.

The *C*. *reinhardtii* genome encodes multiple proteins with the characteristics of neuropeptide precursors, along with proteins that resemble the endo- and exoproteases known to produce PAM substrates from inactive precursors in animals [13]. Our previous analysis identified 771 *C*. *reinhardtii* proteins with an N-terminal signal peptide; many of these exhibit the key features defining neuropeptide precursors (Fig 1A). Examination of those with subtilisin-like prohormone convertase cleavage sites [(K/R)(K/R)] identified 331 with potential amidation sites. Furin-like cleavage sites [RX(K/R)R] were identified in 224 proteins, with 33 including potential amidation sites. Forty-nine proteins have a C-terminal Gly, allowing amidation to occur without the need for any protease, and 24 have a C-terminal Gly(K/R)_n_ sequence, requiring only carboxypeptidase B–like activity to generate a PAM substrate (Fig 1A) [13]. The *C*. *reinhardtii* genome encodes 21 subtilisin-like S8 domain–containing proteases; based on the predicted presence/absence of a signal peptide and/or a transmembrane helix (TMH), 14 might be expected to play a role in the secretory pathway (Fig 1B) [13]. A gene annotation screen identified 146 plasma membrane receptor–related proteins; these were grouped on the basis of their predicted structure and function (Fig 1C and S1 Table). In addition, *C*. *reinhardtii* contains active PAM, which plays a key role in ciliogenesis [12, 14]. Thus, all of the components needed for the production of secreted signaling peptides are present in *C*. *reinhardtii* even though secretory granules, which are used to store bioactive peptides in species as diverse as *Trichoplax*, *Drosophila*, and humans, have not been observed.

### Expression of CrPAM protein and enzymatic activity is regulated during the sexual life cycle

Under favorable conditions, haploid *C*. *reinhardtii* reproduce asexually; a series of mitotic divisions are followed by hatching from the mother cell wall [25,26]. Under conditions of nutrient deprivation, mitotic division ceases and vegetative cells differentiate into mating type minus or plus gametes. Gametes express mating type–specific genes that allow them to recognize each other, fuse, and form quadriflagellate zygotes [27,28]. Previously published transcriptomic studies of specific stages in this complex process revealed an increase in CrPAM mRNA levels as vegetative cells differentiated into resting gametes. Transcript levels rose after lysin treatment of resting gametes, which removes the cell wall, and fell dramatically after exposure to dibutyryl (db)–cAMP, which bypasses the initial cilia-dependent recognition and signaling steps (S1 Fig) [29,30].

**Fig 2:**
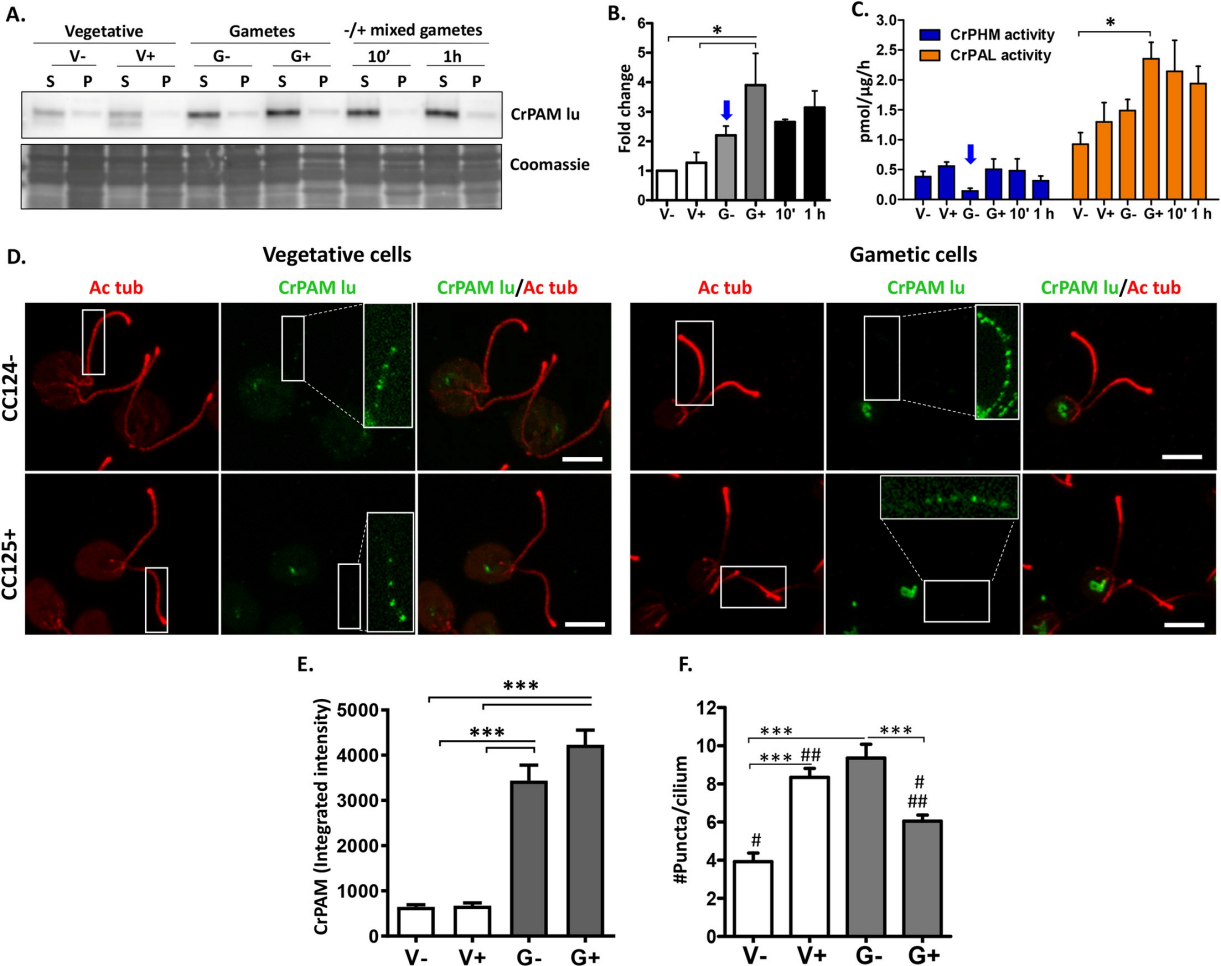
CrPAM expression varies during the sexual life cycle. **A.** Immunoblot of detergent soluble (S) and insoluble (P) fractions of minus and plus vegetative cells (V−, V+), minus and plus resting gametes (G−, G+), and samples taken 10 minutes and 1 hour after mixing minus and plus gametes. Equal amounts of protein (30 μg) were loaded (Coomassie-stained segment is shown) and affinity-purified luminal domain antibody was used to identify CrPAM. **B.** Quantification of immunoblot data revealed that CrPAM protein levels were significantly higher (*P = 0.018) in plus gametes compared with minus and plus vegetative cells; mean ± SEM (*n* = 4). **C.** CrPHM and CrPAL specific activities in detergent soluble fractions used for immunoblot analysis. PAL activity was higher in plus gametes than in minus vegetative cells (*P = 0.011); mean ± SEM (*n* = 5). **D.** Maximal projection confocal images of minus and plus vegetative cells and gametes stained with antibodies against acetylated tubulin (Ac tub, red) and the CrPAM luminal domain (CrPAM lu, green). The boxed regions are contrast enhanced and enlarged in the CrPAM channel to reveal the punctate CrPAM staining. Scale bar = 5 μm. **E.** Quantification of CrPAM immunofluorescence integrated intensity in the cell bodies of vegetative cells and resting gametes. CrPAM intensity was higher in gametes than in vegetative cells (***P < 0.0001; *n* = 20–24 cells for each cell type; one-way ANOVAs). **F.** Quantification of the number of PAM-positive puncta in cilia (#, ##, **P < 0.001, ***P < 0.0001; 30–70 cilia were analyzed for each group ± SEM). The underlying numeric data for this figure can be found in S1 Data. PAL, peptidyl-α-hydroxyglycine α-amidating lyase; PAM, peptidylglycine α-amidating monooxygenase; PHM, peptidylglycine α-hydroxylating monooxygenase.

To explore the possibility that CrPAM and its amidated products might play a role in sexual reproduction, we evaluated PAM expression in vegetative cells and in resting and mating gametes (Fig 2A–2F). None of the approaches used revealed a difference in CrPAM expression between minus and plus vegetative cells. Based on immunoblot analysis, PAL specific activity, and immunofluorescence intensity, levels of CrPAM expression in plus gametes exceeded levels in vegetative cells. Levels of CrPAM protein and PAL specific activity in mating cells were indistinguishable from levels in plus gametes.

While changes in CrPAL specific activity paralleled changes in CrPAM protein, changes in CrPHM specific activity did not (Fig 2C). Both enzyme assays utilize a concentration of peptide substrate that is far below the K_m_, and thus the measured rate varies directly with substrate concentration; in lysates of vegetative cells (Fig 2C), as in assays of rodent tissue, PAL specific activities exceeded PHM specific activities by a factor of about three, reflecting its faster turnover rate [10]. The most striking change in this ratio was observed in minus gametes; despite an increase in PAM protein levels in minus gametes versus minus vegetative cells, PHM specific activity in minus gametes declined substantially (Fig 2B and 2C; blue arrows), suggesting that additional mechanisms are used to control PHM.

Previous studies localized CrPAM to the Golgi complex and to cilia in vegetative cells of both mating types [12]. The subcellular localization of CrPAM in the Golgi and cilia of resting gametes was evaluated using affinity-purified antibody to its luminal domain and compared to vegetative cells (Fig 2D); PAM expression was significantly higher in gametes than in vegetative cells (Fig 2D and 2E). PAM-positive puncta were visible along the ciliary length of both minus and plus gametes (insets in Figs 2D and S2A). In vegetative cells, CC125 plus cilia had more PAM-positive puncta than CC124 minus cilia; in contrast, PAM-positive ciliary puncta were more numerous in CC124 minus than in CC125 plus gametes (Fig 2F), but the intensity was not significantly different (S2B Fig). CrPAM-positive puncta of high intensity were more common in gametes than in vegetative cells (S2B Fig). Taken together, our data suggested that CrPAM might play a role in sexual reproduction.

### Ciliary ectosome composition is unique and developmentally regulated

Vegetative and gametic *C*. *reinhardtii* release bioactive ectosomes from their cilia [20,22,24]. Vegetative lytic enzyme 1 (VLE1), a subtilisin-like protease required for hatching, is released in daughter cell ectosomes [20], and the agglutinins essential for ciliary adhesion are present in ectosomes released by mating gametes [24,31]. Since mammalian PAM is found in the intraluminal vesicles of multivesicular bodies (MVBs) and in exosomes/extracellular vesicles purified from blood, saliva, and urine [32–35], we hypothesized that CrPAM would be released in ciliary ectosomes.

**Fig 3:**
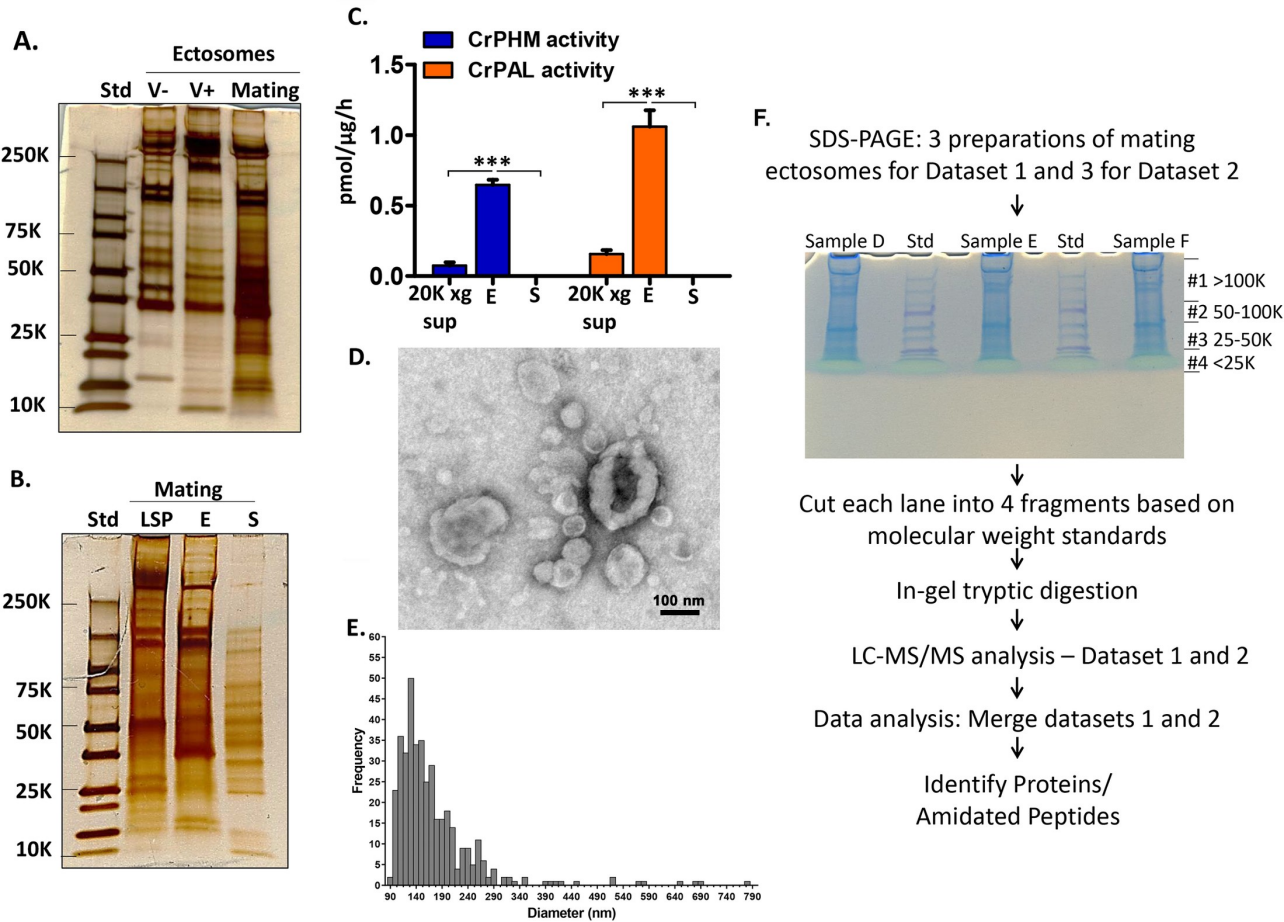
Biochemical characterization of ectosome-rich pellets. **A.** Comparison of ectosome-rich pellets (2 μg protein/lane) prepared from vegetative cells incubated for 4 hours and from gametes mixed and allowed to mate for 1 hour. Proteins were visualized using silver stain. Std, molecular weight markers. **B.** Comparison of proteins in the mating gamete low-speed pellet (LSP, 2 μg protein), ectosome-rich pellet (E, 2 μg protein), and soluble secretome (S, 40 μL corresponds to approximately 80 μg cell protein); proteins were visualized using silver stain. **C.** CrPHM and CrPAL activities were measured in the 20,000*g* supernatant (20K*g* sup), ectosome-rich pellet (E), and soluble secretome (S); specific activities are the average of three independent experiments ± SEM (***P < 0.0001; one-way ANOVAs). **D.** Negatively stained electron microscope image of the ectosome-rich pellet prepared from 1-hour mating medium of mixed minus and plus gametes. **E.** Histogram illustrating the size distribution of isolated ectosomes (mean ± SEM = 170 ± 14 nm; *n* = 400). **F.** Mating ectosome-rich pellets prepared from biological triplicates two independent times were subjected to mass spectrometric analysis. All six samples were separated by SDS-PAGE; after visualization with colloidal Coomassie, each gel lane was sliced into four fragments as shown. Proteins and amidated peptides were identified by mass spectrometric analysis (S2 Table). The underlying numeric data for this figure can be found in S1 Data.

Ectosome-enriched pellets were prepared from vegetative cells and from mating gametes to test this hypothesis. Since ciliary adhesion during mating elicits an increase in the release of ectosomes [24,36], vegetative cells were incubated for 4 hours and mating gametes for 1 hour. In 1 hour, mating gametes released 2.5% ± 0.2% (mean ± SEM) of their total cellular protein in ectosomes; in 4 hours, minus and plus vegetative cells released 0.12% ± 0.06% and 0.44% ± 0.17% of their total cellular protein, respectively, in ectosomes (S3A Fig). SDS-PAGE of equal amounts of protein from ciliary ectosomes released by minus and plus vegetative cells and mating gametes revealed distinctly different patterns (Fig 3A).

To assess the success of the differential centrifugation protocol used to enrich ectosomes, we compared samples prepared from mating gametes, revealing major differences (Fig 3B). Strikingly, assays for PHM and PAL activity identified both enzyme activities in the 20,000*g* supernatant (20K*g* sup) and ectosomes but their absence from the soluble secretome (Fig 3C). Negative stain electron microscopy revealed a heterogeneous collection of approximately 100- to 400-nm diameter vesicles in the ectosome-rich pellet (Fig 3D and 3E); many of the vesicles had the round/cup-shaped morphology characteristic of ectosomes.

### Proteomic analysis of the mating ectosome-rich pellet

Since we identified PAM enzyme activity in mating ectosomes but not in the soluble secretome, we turned to mass spectrometry to search for peptide precursors, subtilisin-like endoproteases, and potential peptide receptors in the mating ectosome-rich pellet. SDS-PAGE was used to separate each sample into four fractions (Fig 3F). The complete dataset, which includes only proteins identified in at least four of the six biological replicates, appears in S2 Table. CrPAM was identified in all six mating ectosome samples, with an average coverage of 11% ± 2.8% (mean ± SEM). A total of 247 signal peptide–containing proteins (including CrPAM) were identified (Fig S3B and S3 Table); the 29 most abundant accounted for 55% of total spectral counts from this protein class (S4 Table). Proteases and ER/Golgi proteins each accounted for about a tenth of the signal peptide–containing ectosomal proteins. Numerous membrane proteins were also identified (S5 Table) and a comparison of signal peptide–containing proteins found in ectosomes and/or the secretome appears in S6 Table.

**Fig 4:**
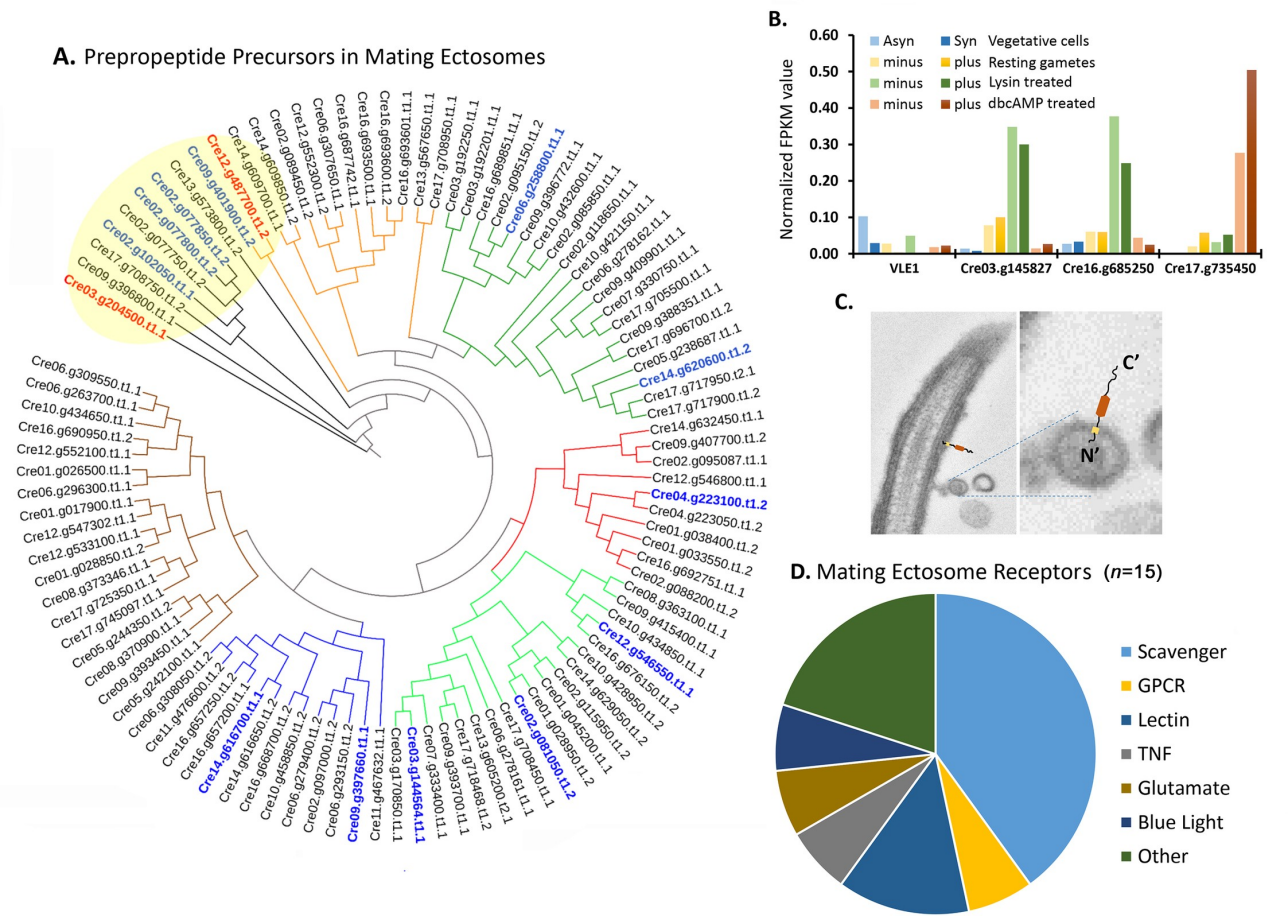
Proteomic analysis of mating ectosome-rich pellets reveals the presence of peptidergic signaling machinery. **A.** Predicted prepropeptides (S7 Table) identified in mating ectosomes were aligned using CLUSTALW. A rooted phylogenetic tree was generated using UPGMA hierarchical clustering; groupings are delineated by colored nodes. The Cre03.g204500 chemotactic peptide precursor and the most abundant amidated product precursor (Cre12.g487700) are indicated in red; the twelve most abundant precursors are shown in blue. **B.** Transcriptomic analysis of the four subtilisin-like Type II membrane endoproteases identified in mating ectosomes (data from [30]). The underlying numeric data for this figure can be found in S1 Data. **C.** Electron micrograph of an ectosome budding from a *C*. *reinhardtii* cilium. The orientation expected of a Type II membrane protein is illustrated. **D.** Fifteen of the 146 *C*. *reinhardtii* receptors (S1 Table) were identified in mating ectosomes. Asyn, asynchronous; db, dibutyryl; FPKM, fragments per kilobase of transcript per million mapped reads; GPCR, G protein–coupled receptor; Syn, synchronous; TNF, tumor necrosis factor; UPGMA, unweighted paired group method with arithmetic mean; VLE1, vegetative lytic enzyme 1.

Bioinformatics identified 771 potential prepropeptides in the *C*. *reinhardtii* genome (Fig 1A, [13]); strikingly, 99 of these were found in the mating ectosome-rich pellet, 73 of which contain potential amidation sites (S7 Table); several amidated products are discussed in the following section. Phylogenetic analysis placed these potential prepropeptides into seven broad groups, with four of the most abundant ones (highlighted in blue) closely related to each other (Fig 4A; yellow shading); three of these four have C-terminal amidation sites (Fig 4A and S7 Table).

A total of 33 proteases (23 signal peptide–containing) were identified in the mating ectosome-rich pellets. Matrix metalloproteinases (MMPs), which are implicated in extracellular matrix (ECM) degradation and growth factor activation [37,38], were the most numerous (S8 Table). In addition to VLE1 (Cre01.g049950), three subtilisin-like prohormone convertases, which would be expected to cleave at the C-terminal side of pairs of basic residues [39], were present in the mating ectosome-enriched pellet (Fig 4B). Interestingly, all four identified subtilisin type proteases are Type II membrane proteins; their predicted topology in cilia and ectosomes is illustrated in Fig 4C. Based on published transcriptomic data, VLE1, which resembles prohormone convertase PC7 (PCSK7), is most highly expressed in vegetative cells [29,30], consistent with its essential role in mother cell wall degradation [20]. Transcripts encoding Cre03.g145827 and Cre16.g685250, which resemble PCSK4 (PC4) are abundant in lysin-treated gametes of both mating types (Fig 4B), [30]. Transcripts encoding Cre17.g735450, which resembles PCSK2 (PC2) and PCSK6 (PACE4), are most plentiful in db-cAMP–treated plus gametes [29,30]. The unique regulatory patterns observed for the different subtilisin-like prohormone convertases suggest that each could be used to target different substrates at specific developmental stages (Fig 4B and S8 Table).

Six scavenger receptors, a GPCR, two lectin receptor–related proteins, a tumor necrosis factor (TNF) receptor–related protein, one ionotropic glutamate receptor, the flagellar blue light receptor, and three other receptor–related proteins were identified in mating ectosomes (Fig 4D and S1 Table). Different receptors showed distinctly different transcriptomic expression patterns. Some scavenger receptors were most highly expressed in resting minus gametes, while the only identified GPCR was most highly expressed in lysin-treated gametes (S1 Table) [29,30].

### Identification of amidated products in ectosome-rich pellets

**Fig 5:**
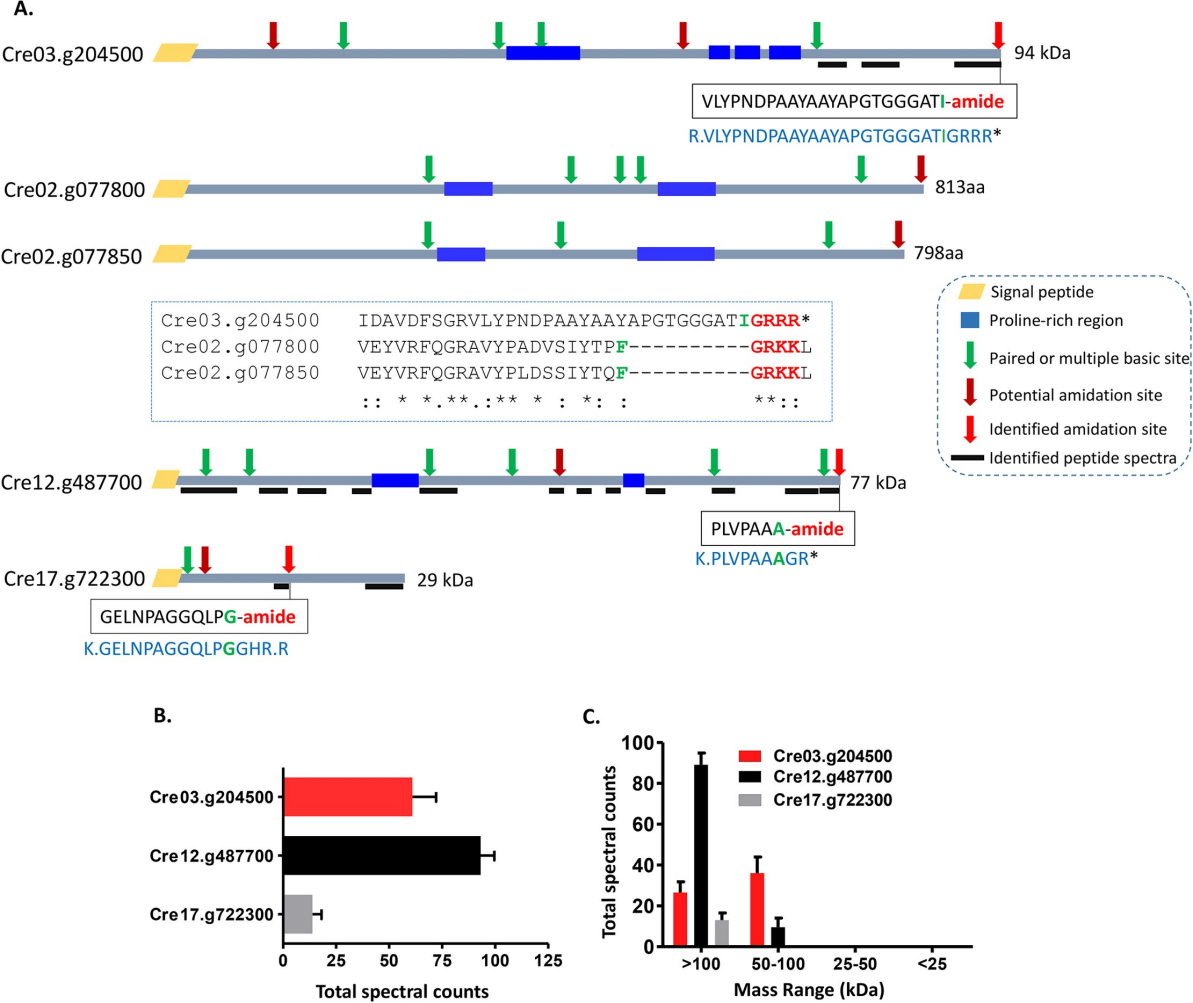
Identification of amidated products in mating ectosome-rich pellets. **A.** Sequences of the three amidated tryptic peptides identified are shown in boxes; the amidated residue identified is indicated in green. For each identified amidated tryptic peptide, a schematic diagram of the protein that could have generated it is shown; the sequence of each of these larger proteins (shown in blue) includes a cleavage site and a glycine residue. Thick black lines mark tryptic peptides that were identified throughout the proprotein. The domain organization and potential C-terminal amidation sites of Cre02.g077800 and Cre02.g077850, signal peptide–containing prepropeptide precursors (S7 Table) that resemble amidated protein precursor Cre03.g204500, are shown; neither of these potential amidated peptides was detected. **B** and **C.** Total spectral counts (*n* = 6; ± SEM) for the identified amidated protein precursors and the average number of spectral counts in gel slices encompassing proteins of different apparent molecular weight ranges are indicated. The underlying numeric data for this figure can be found in S1 Data. aa, amino acid.

When our mass spectrometry datasets were screened for amidation using the “Gly-loss + amide” filter, three amidated peptides were identified in all six samples (Figs 5 and S3C). The sequences of these amidated peptides and a schematic of the proteins from which they could be generated are shown in Fig 5A. Carboxypeptidase B–like trimming of intact Cre03.g204500, which terminates with–GRRR*, and intact Cre12.g487700, which terminates with–GR*, would yield PAM substrates. Endoproteolytic cleavage of Cre17.g722300, followed by exoproteolytic removal of–Arg and–His, would generate a PAM substrate (Fig 5A); carboxypeptidase B–like enzymes can remove C-terminal His residues [40]. All three proproteins contain multiple paired basic residues (KR/RR) and potential amidation sites (Fig 5A). Multiple sequence alignment revealed significant homology extending throughout the length of the three prepropeptides related to Cre03.g204500, with similarities in mass, the location of proline-rich regions, and cleavage sites; Cre02.g077800 and Cre02.g077850, which are immediately adjacent in the genome, both contain amidation sites close to their C termini (Fig 5A). Cre12.g487700 is homologous to Cre09.g401900, one of the 10 most abundant prepropeptides identified in mating ectosomes (Fig 4A and S7 Table).

Based on spectral counts, Cre03.g204500 and Cre12.g487700 were more highly expressed than Cre17.g722300 (Fig 5B). The gel slices that yielded these three amidated peptides contained proteins larger than 50 kDa, suggesting that the intact proteins were amidated (Fig 5C); even Cre17.g722300, a 29-kDa protein, was only recovered from the high molecular weight fractions. Published transcriptomic datasets for genes expressed in vegetative cells and during the *C*. *reinhardtii* sexual cycle revealed distinctly different expression patterns for CrPAM and these three substrates [29,30] (S1 Fig). Based on its prevalence, enrichment in mating type plus gametes, and further transcriptomic enhancement following activation with db-cAMP, we focused on the amidated peptide produced from Cre03.g204500. Although the coding region predicted for Cre03.g204500 lacks a signal sequence, an in-frame signal sequence is encoded in the 5′-untranslated region, suggesting that the current gene model is incorrect and that the coding region includes an additional 150 bp upstream of the predicted start site. Interestingly, Cre03.g204500 is a member of an extended family of peptide precursor-like genes that includes an 11-member gene cluster on chromosome 8 (S4A–S4C Fig).

### GATI-amide acts as a gamete chemotactic modulator

**Fig 6:**
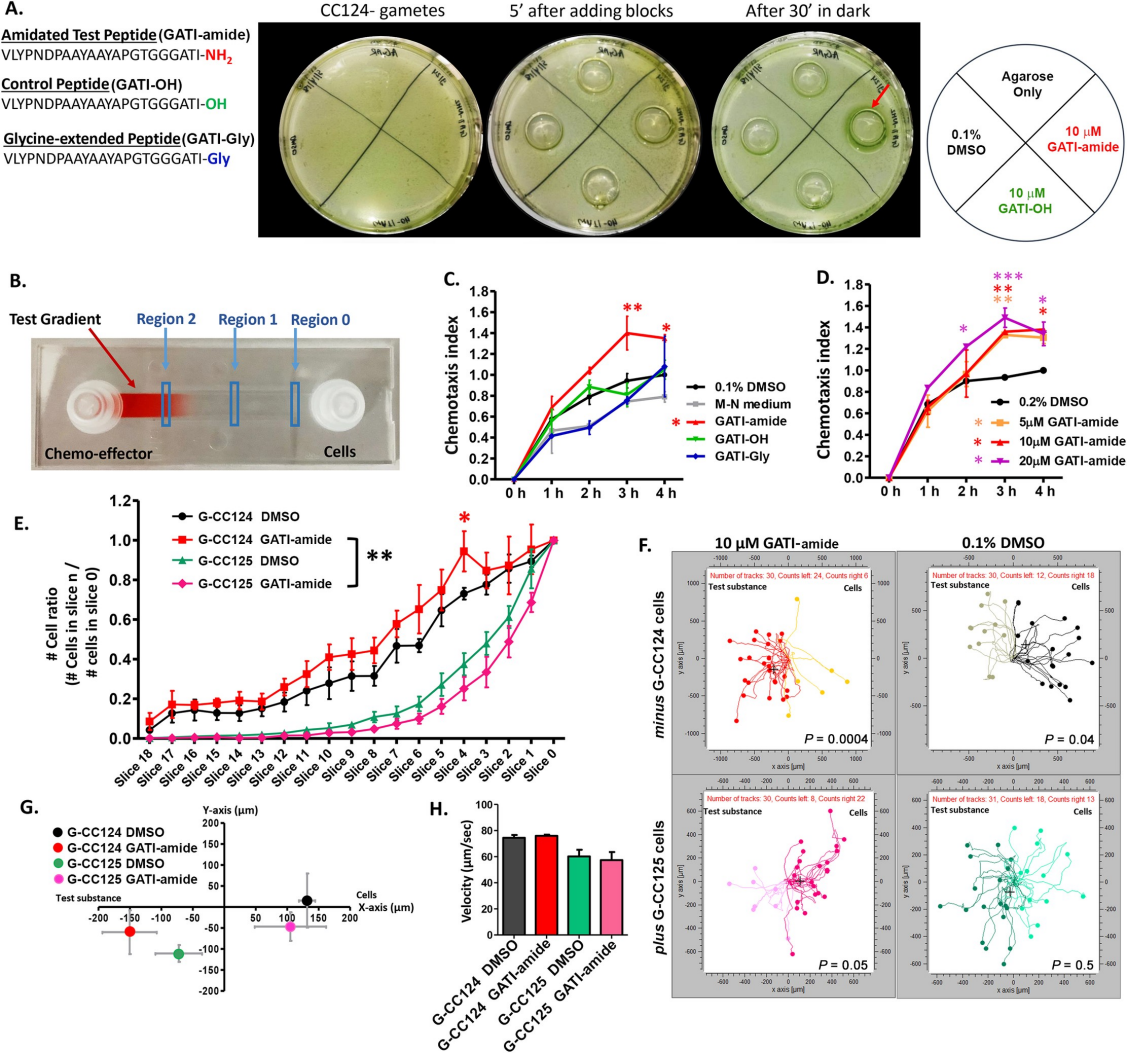
GATI-amide acts as a chemotactic modulator for *C*. *reinhardtii* gametes. **A. Agarose block assay.** CC124 minus gametes were uniformly spread on the surface of a Petri dish containing 1% agar in M-N medium. Agarose blocks containing 10 μM GATI-amide, 10 μM GATI-OH, M-N medium, or M-N medium containing 0.1% DMSO were placed onto the Petri dish; after 30 minutes in the dark, minus gametes had accumulated around the agarose block containing GATI-amide (red arrow). Similar results were obtained in three independent experiments. **B. Microfluidic chemotaxis assay.** Microfluidic channel slides were used for chemoattractant gradient formation. An image of a gradient of red food dye is shown. For each treatment, the number of cells in regions 0, I, and 2 were counted over time. **C. Chemotaxis assay of minus gametes in microchannel slide**. The chemotaxis index (CI) was measured over a period of 4 hours in response to 10 μM GATI-amide, GATI-OH, GATI-Gly, M-N medium, and vehicle control. The CI in the presence of GATI-amide was significantly higher at 3 hours (**P < 0.01) and 4 hours (*P < 0.05) compared to 0.1% DMSO. **D. Dose dependence of the response of minus gametes to GATI-amide**. A 4-hour incubation period was used; 0.2% DMSO was used as the vehicle control to accommodate the use of 20 μM peptide. The CI of 5 μM GATI-amide was significantly higher than the control at 3 hours (**P < 0.01); for 10 μM GATI-amide, the CI was significantly higher at 3 hours (**P < 0.01) and 4 hours (*P < 0.05), while for 20 μM GATI-amide it was significantly higher at 2 hours (*P < 0.05), 3 hours (***P < 0.01), and 4 hours (*P < 0.05). The only CI to ever exceed 1.00 was for GATI-amide. **E. Chemotactic response**. The response of minus and plus gametes to 10 μM GATI-amide and 0.1% DMSO. The number of cells for each treatment was counted along the entire channel length, and the ratio of the number of cells in each slice/number of cells in slice 0 (n_slice X_/n_slice 0_) was plotted for the 4-hour time point. The response of gametic CC124 cells to GATI-amide was significantly (**P < 0.001) different from gametic CC125 cells. Data are the average of three independent experiments ± SEM. **F. Trajectory plots**. Plots are shown for minus and plus gametes. Data are representative of three independent experiments; all plots shown are from one experiment. **G. Population response.** The center of mass was calculated for each population from trajectory plots like those shown in **F**. Results are the average of three independent experiments ± SEM. **H. Motile behavior.** The swimming velocities of minus and plus gametes were not altered by the presence of the amidated peptide. Results are the average of three independent experiments ± SEM. One-way and two-way ANOVAs were used as appropriate. The underlying numeric data for this figure can be found in S1 Data. GATI-amide, VLYPNDPAAYAAYAPGTGGGATI-amide; M-N medium, gametic medium.

The amidated peptides studied in basal metazoans affect a variety of processes, including ciliary motility, shape, movement, and velocity [6]. Since *C*. *reinhardtii* exhibit positive chemotaxis towards nitrogen sources (NH_4_Cl), carbon sources (HCO_3_^−^), and NaCl [41], we evaluated the ability of the amidated peptide produced from Cre03.g204500 to act as a chemoattractant. The GPCRs that bind amidated peptides generally recognize short peptides that terminate with the amidated residue. Instead of expressing the 94-kDa Cre03.g204500 protein, we therefore had three peptides synthesized: VLYPNDPAAYAAYAPGTGGGATI-amide (abbreviated GATI-amide), its putative precursor (abbreviated GATI-Gly), and a peptide in which Ile-amide was replaced with Ile (abbreviated GATI-OH) (Fig 6A). The higher levels of PAM protein and Cre03.g204500 mRNA in plus gametes led us to test the effects of GATI-amide and GATI-OH on minus gametes using an agarose block assay (Fig 6A). After 30 minutes in the dark (to negate any phototactic effects), minus gametes accumulated around the agarose block containing GATI-amide but were not attracted to the agarose blocks containing control peptide (GATI-OH), gametic medium (M-N medium), or 0.1% DMSO (vehicle control). GATI-amide did not act as an attractant in similar experiments with plus gametes.

For a more readily quantifiable assessment of chemotaxis, we utilized slides with an imaging channel (50 mm × 5 mm × 0.2 mm) and access chambers at both ends that facilitated gradient formation (adapted from Choi and colleagues, 2016). The gradient present 1 hour after injecting 10 μL of a low molecular weight dye into the imaging chamber via the chemo-effector port and removing the same volume from the cell port is shown (Fig 6B). Time-lapse fluorescence imaging of a gradient formed by injecting 10 μL of a 1 μM stock of a fluorescein isothiocyanate (FITC)–tagged peptide (molecular weight, 1,542 Da) demonstrated its long-term stability (S5 Fig).

We first evaluated the chemotactic response of minus gametes (introduced into the chamber labeled “Cells”) to gradients formed from equal concentrations of GATI-amide, GATI-OH, or GATI-Gly introduced via the “Chemo-effector” chamber; the vehicle control was M-N medium containing 0.1% DMSO (Fig 6C). The numbers of cells identified in Region 1 and Region 2 were determined as a function of time, allowing calculation of the chemotaxis index (CI) [(number of cells in region 2 ÷ number of cells in region 1) ÷ (same ratio for cells kept 4 hours in DMSO vehicle control)]. After 3–4 hours, minus gametes were more attracted to GATI-amide than to any of the controls (Fig 6C). We next tested the effect of GATI-amide concentration on the chemotactic response of minus gametes (Fig 6D). A chemotactic response to 20 μM GATI-amide was apparent at 2 hours; by 3 and 4 hours, the chemotactic responses to 5, 10, and 20 μM GATI-amide were similar, suggesting saturation of the putative amidated peptide receptor.

To provide a more comprehensive analysis of the chemotactic response, we took 19 images, covering the length of each channel, and determined the cell number in each image (Fig 6E). Data for minus and plus gametes exposed to gradients generated from 0.1% DMSO or 10 μM GATI-amide for 4 hours are shown in Fig 6E–6H. Minus gametes (CC124) exhibited a positive chemotactic response to GATI-amide versus DMSO. In contrast, plus gametes (CC125) exhibited a negative chemotactic response to GATI-amide versus DMSO at 4 hours (Fig 6E).

Since plus and minus gametes showed different responses to GATI-amide, movements of both gametes in each condition were followed. As *C*. *reinhardtii* move rapidly (100–150 μm/second), we tracked cells for 10 seconds at regions 0 and 1 of the microchannel slides using 0.5-second time intervals. As show in Fig 6F, more minus gametes moved to the left (i.e., towards or up the peptide gradient) than to the right (away from or down the peptide gradient) in the gradient formed using 10 μM GATI-amide, while the opposite result was observed with 0.1% DMSO, the vehicle control. With plus gametes, cells were repelled by the amidated peptide and moved toward the vehicle control (Fig 6F lower panel).

The center of mass (COM) was calculated for each group of tracked cells (Fig 6G), providing a population-based readout of chemotaxis. The G124 minus gametes showed displacement of their COM toward the amidated peptide and away from the vehicle control (Fig 6G). In contrast, the COM for G125 plus gametes moved away from the amidated peptide and towards the vehicle control (Fig 6G). The differences in chemotactic behavior of minus and plus gametes were not a response to changes in ciliary-generated propulsive force, as the swimming velocity of minus and plus gametes was not altered by the presence of either the amidated peptide or DMSO (Fig 6H).

### Developmentally regulated release of CrPAM in ciliary ectosomes

**Fig 7:**
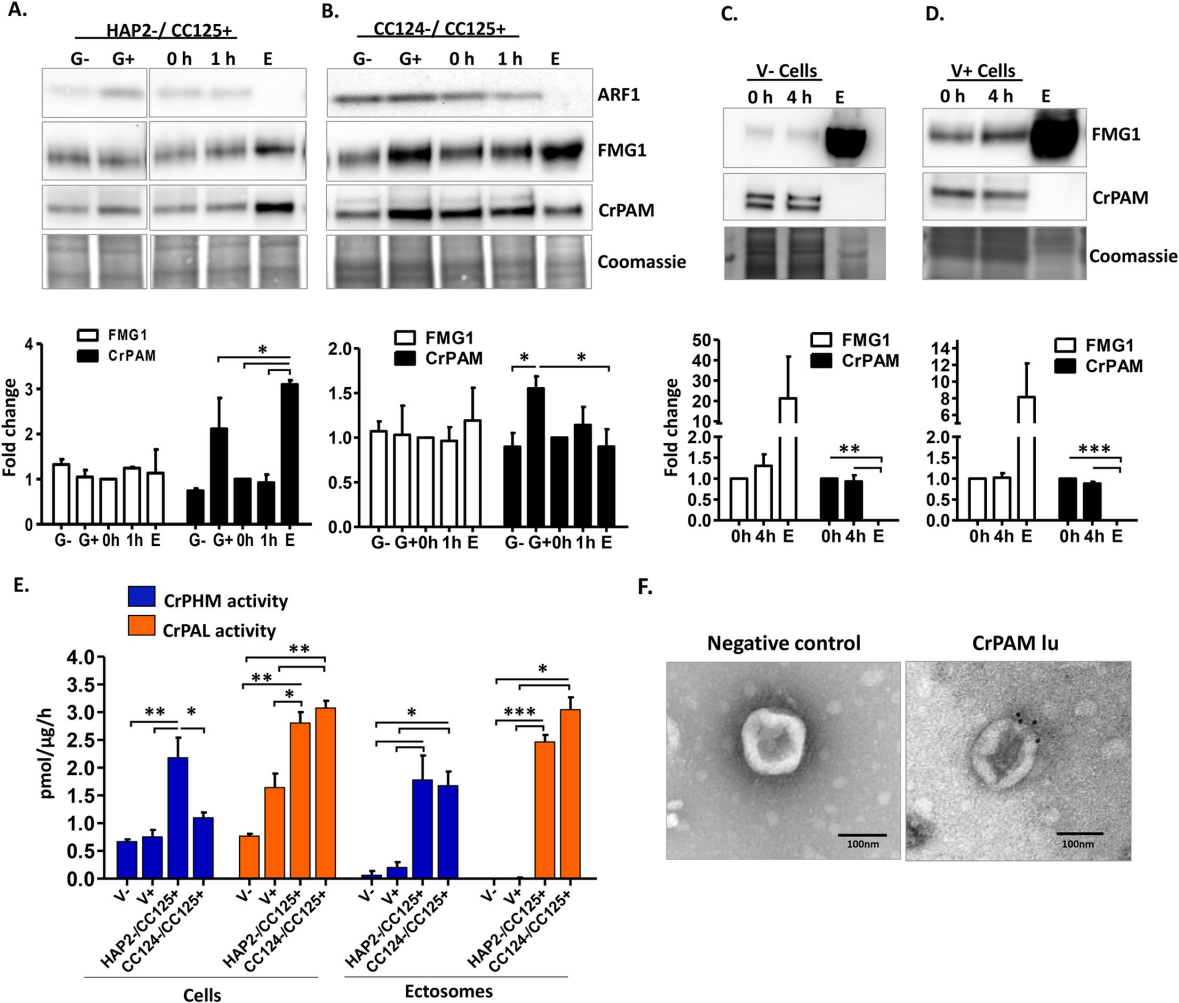
Release of CrPAM in ciliary ectosomes is developmentally regulated. **A.** HAP2 minus and CC125 plus gametes, mating cells (0- or 1-hour), and ectosome-rich pellets prepared from the 1-hour media were analyzed. Equal amounts of protein (30 μg) were fractionated and subjected to immunoblot analysis for ARF1, FMG1, and CrPAM. Quantification of FMG1 and CrPAM protein levels is shown in the lower panels; results are the average of two independent experiments—error bars indicate the range. **B.** Cell lysates and ectosomes prepared from wild-type CC124−/CC125+ gametes were analyzed as described for panel **A**. Results are the average of six independent experiments; error bars indicate ± SEM. Asterisks indicate a statistically significant difference between two groups (*P < 0.01). **C.** and **D.** Immunoblot analysis of cells and ectosomes harvested from vegetative CC124− (**C**) and CC125+ (**D**) cells; quantification of FMG1 and CrPAM levels is shown below. Results are the average of three experiments; error bars indicate ± SEM. CrPAM and FMG1 levels in vegetative ectosomes differed significantly from levels in cells (**P = 0.0065, ***P < 0.0001). **E.** CrPHM and CrPAL activities were assayed in cells and ectosomes released by vegetative CC124− and CC125+ cells, mating HAP2−/CC125+ cells, and mating CC124−/CC125+ cells. Both activities were significantly higher in ectosomes released by mating gametes (*P < 0.01, **P < 0.001, ***P < 0.0001; one-way ANOVAs). **F.** Immunogold-electron microscopy negative stain image showing localization of CrPAM on mating ectosomes with antibody against CrPAM luminal domain. Negative control, ectosomes incubated with gold-tagged secondary antibody alone. The underlying numeric data for this figure can be found in S1 Data. ARF1, ADP ribosylation factor 1; FMG1, flagellar membrane glycoprotein 1.

During sexual reproduction in *C*. *reinhardtii*, the cilia of mating type minus and plus gametes adhere to each other, triggering formation of mating structures and subsequent cell fusion. We took advantage of mutations in HAP2, a mating type minus–specific protein, that prevent cell fusion, prolonging ciliary release of mating ectosomes [36]. Ectosomes were prepared from fusion defective HAP2 minus gametes mixed with CC125 plus gametes (Fig 7A). As expected, release of ectosomal protein was elevated (4.6% ± 0.4% of cell protein/hour) compared to release by wild-type gametes. We then evaluated the specificity with which different proteins entered ectosomes (Fig 7A). Flagellar membrane glycoprotein 1 (FMG1), which has been identified in vegetative ectosomes [22,24], was present but not generally enriched, although levels were somewhat variable in the mating ectosome-rich pellet. ADP ribosylation factor 1 (ARF1), an ER-Golgi associated protein, was largely absent from the ectosome-rich pellet. PAM protein levels in plus gametes were again higher than in minus gametes. Most strikingly, PAM protein levels in the mating ectosome-rich pellet were significantly higher than in mating cells harvested after the 1-hour collection period. We also examined mating ectosomes released by wild-type gametes (Fig 7B). FMG1 was again present but not enriched in the mating ectosomes, and ARF1 was again largely excluded. As observed in Fig 1B, CrPAM levels were higher in resting plus gametes than in resting minus gametes. Levels of CrPAM protein were similar in cells mixed for 1 hour and in the ectosomes released during this 1-hour period (Fig 7B).

We next examined ectosomes released by vegetative cells of both mating types (Fig 7C and 7D). For both FMG1 and PAM, striking differences were observed in the extent to which they were concentrated in ectosomes. Levels of FMG1, which was not enriched in mating ectosomes, were highly enriched in ectosomes released by both minus CC124 and plus CC125 vegetative cells (Fig 7C and 7D). In contrast, PAM protein, which was present in mating ectosomes, was excluded from ectosomes released by vegetative cells. Enzyme assays were used to compare the specific activities of PAM in cell lysates and ectosomes (Fig 7E). PHM and PAL activities in vegetative ectosomes were essentially indistinguishable from background; in contrast, mating ectosomes contained high levels of both activities (Fig 7E).

Localization of CrPAM on mating ectosomes was further confirmed by immuno-electron microscopy using an affinity-purified antibody against the luminal domain of CrPAM and a gold-tagged secondary antibody (Fig 7F). Ectosomes incubated only with gold-conjugated antibody to rabbit immunoglobulin lacked gold particles. The differences observed in PAM and FMG1 levels in ectosomes prepared from mating versus vegetative cells suggested that a better understanding of their trafficking could shed light on the mechanisms that control ciliary protein levels.

### Rapid redistribution of CrPAM and release in mating ectosomes

**Fig 8:**
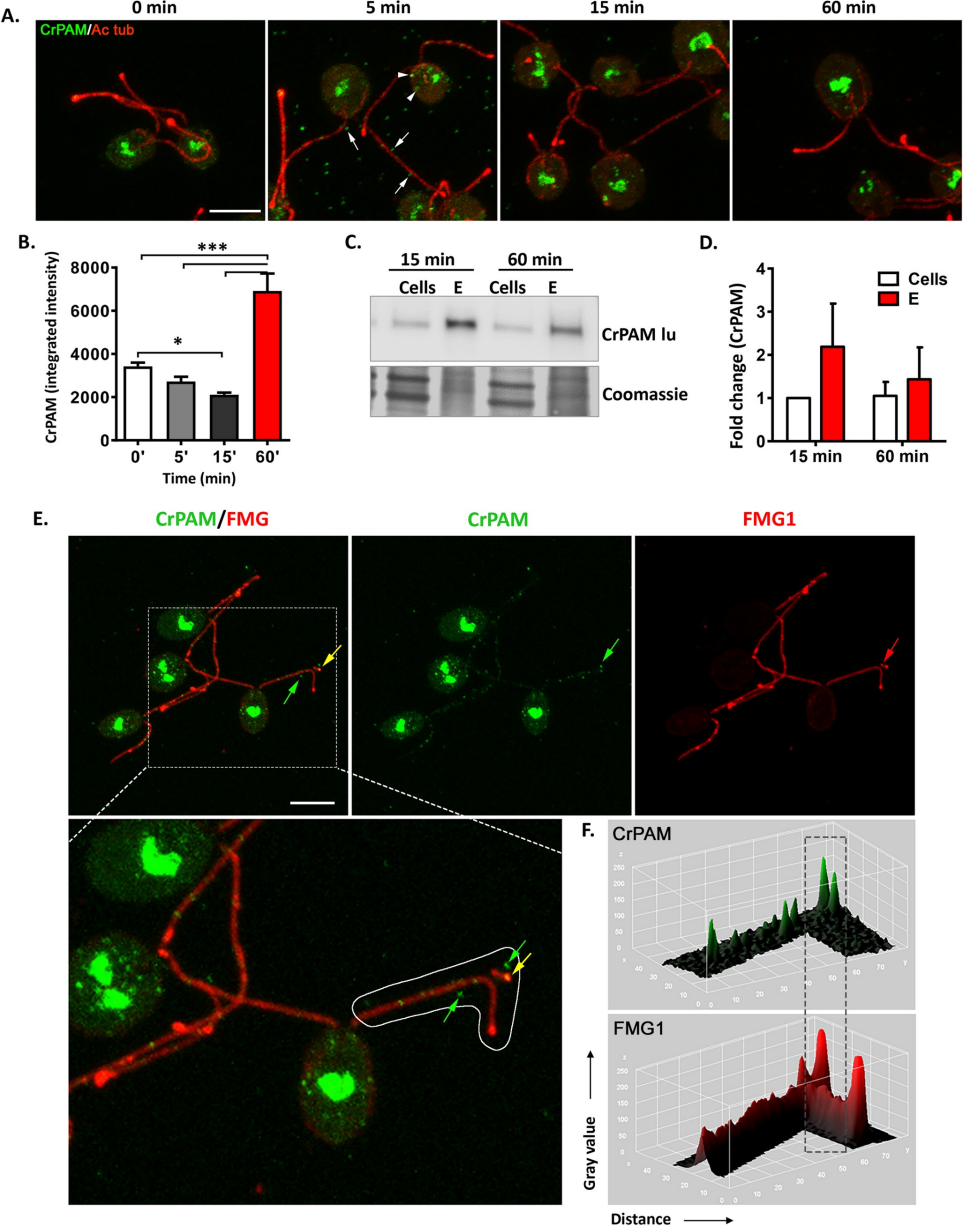
CrPAM is rapidly released in mating ectosomes. **A**. Maximal projection confocal images of minus and plus gametes fixed at the indicated times after mixing; permeabilized cells were stained using antibodies against the luminal domain of CrPAM (green) and Ac tub (red). CrPAM-positive ectosomes are marked by white arrows, and CrPAM-positive vesicles in the cell body are indicated by white arrowheads. **B.** Quantification of cell body CrPAM staining as a function of time. Data are averages of 28–37 cells ± SEM (*P < 0.01, ***P < 0.0001). **C.** Cells and ectosome-rich pellets (20 μg protein) prepared 15 and 60 minutes after gametes were mixed were subjected to immunoblot analysis using the CrPAM luminal antibody. **D.** Data for CrPAM levels in cells and their ectosome-rich pellets 15 and 60 minutes after mixing were quantified. Data are the average of duplicates, with error bars indicating the range. **E.** Maximal projection confocal images of cells fixed 5 minutes after minus and plus gametes were mixed, showing release of ciliary ectosomes; arrows mark puncta positive for CrPAM (green), FMG1 (red), or both FMG1 and CrPAM (yellow) (see expanded image). Scale bar = 5 μm. **F.** Surface intensity plots of CrPAM and FMG1 staining along the cilium outlined in panel **E**. The underlying numeric data for this figure can be found in S1 Data. Ac tub, acetylated tubulin; FMG1, flagellar membrane glycoprotein 1.

In vegetative cells, CrPAM is largely localized to the Golgi region, with less than 10% recovered from cilia [12,14]. Fertilization proceeds rapidly; zygotes are formed within minutes after the gametes are mixed and gamete fusion is largely completed within 30 minutes [42]. To evaluate changes in CrPAM localization during mating, cells were fixed immediately (0 minutes) or 5, 15, and 60 minutes after mixing gametes. CrPAM and acetylated tubulin (Ac tub) were visualized in permeabilized cells (Fig 8A). An increase in CrPAM-positive vesicles in the cell body was apparent within 5 minutes of mixing; after 60 minutes, vesicular CrPAM staining declined and PAM was largely localized to the Golgi region (Fig 8A). Ac tub staining did not vary with time. Quantification of cell body CrPAM staining revealed a decline after 15 minutes, followed by an increase after 60 minutes (Fig 8B). CrPAM-positive puncta that were not associated with cell bodies or cilia were prevalent in images of cells fixed 5 minutes after mixing (Fig 8A). To determine whether these puncta could represent rapidly released ectosomes, ectosome-rich pellets were prepared from cells 15 and 60 minutes after the initiation of mating (Fig 8C). CrPAM expression was detected in both ectosome-rich pellets (Fig 8C and 8D).

We next compared the trafficking behavior of CrPAM and FMG1 as a function of time after gamete mixing (Figs 8E and S6). In the cell body at 0 minutes, FMG1 was detected at the plasma membrane, forming a ring-like structure (inset in S6 Fig, top left panel). Continuous FMG1 staining was observed along the length of the cilium, with peaks at what appeared to be sites of ectosome release. PAM staining was apparent in ciliary and cytoplasmic puncta; cell body staining was localized to the Golgi region, with no concentration on the plasma membrane (Figs 8E and S6). Non-cell associated CrPAM-positive puncta were most numerous 5 minutes after mixing, and many of these were also positive for FMG1 (Fig 8E). Surface intensity plots for CrPAM and FMG1 staining in cilia and puncta not associated with cells (Fig 8F) distinguished sites of CrPAM/FMG1 co-localization from sites enriched only in PAM or only in FMG1 (Fig 8E). Analysis of these two ectosomal cargo proteins demonstrated that cells release ectosomes that are not all identical. The rapid release of CrPAM in mating ectosomes is consistent with a role for PAM in the cilium-generated signaling events involved in mating.

## Discussion

### Peptidergic signaling is ancient

Secreted peptides play fundamental roles in controlling metabolism, reproduction, memory, and behavior. Genomic studies have revealed striking similarities between the peptide processing machinery present in organisms as diverse as bilateria, cnidarians, ctenophores, placazoans, and sponges [6,43–45]. For example, in *Trichoplax adherens*, which has only six cell types and lacks neurons, transcripts encoding putative prepropeptides that could generate amidated peptides are expressed in different cell populations; exposure to individual peptides elicits unique changes in animal behavior [6]. Similarly, sea urchin eggs release sperm-activating peptide-1, leading to activation of a sperm guanylate cyclase [46], while egg-derived amidated chemotactic peptides attract sperm by binding in a species/genus-specific manner to scavenger receptors localized on the flagellum.

Our bioinformatics analysis revealed the presence of hundreds of prepropeptide-like molecules in the *C*. *reinhardtii* genome. The prepropeptides identified in mating ectosomes fall into clusters of related proteins, suggesting the occurrence of gene duplications, as observed for metazoan prepropeptides. While many metazoan peptide precursors contain multiple copies of the same peptide, *C*. *reinhardtii* prepropeptides do not. It is striking that two of the three amidated products identified in mating ectosomes derived from C-terminal amidation sites [-Gly or–Gly(K/R)(K/R)], eliminating the need for endoproteolytic cleavage. *C*. *reinhardtii* lacks structures resembling the secretory granules that store the products of metazoan prepropeptide processing. Our data indicate that cilia provide an alternate route for bioactive peptide secretion.

As multicellularity allows for dedicated functionality of individual cell types, the specialized secretory cells common in mammals often store and release the products of a single major prepropeptide in response to specific inputs [1,3,12]. In contrast, unicellular *C*. *reinhardtii* may need to secrete many different peptides in response to varying biotic and abiotic challenges (see below) and thus requires more signaling flexibility than provided by the dedicated storage and secretion of a single product.

PAM is expressed at all life cycle stages in *C*. *reinhardtii*, with protein levels and activity selectively increasing during gametogenesis. Treatment of *C*. *reinhardtii* gametes with db-cAMP, bypassing the gamete activation process, down-regulates PAM expression, suggesting complex regulation of peptidergic machinery during algal mating. In rodents, uterine expression of PAM is estrogen-sensitive and gonadotropin hormone releasing hormone (GnRH), an amidated peptide, plays an essential reproductive role [47]. In *Drosophila*, myoinhibitory peptides and the sex peptide receptor function in a female-specific manner [48], while two other neuropeptides inhibit reproductive dormancy [49]. Various neuropeptides also control the activity of silkworm male reproductive organs, regulating seminal fluid movement during copulation [50], while a short amidated peptide (W/RPRP-amide) is responsible for inducing oocyte maturation in hydrozoan jellyfish [51]. Thus, a key reproductive role for peptides amidated by PAM has been broadly conserved.

### Peptidergic signaling machinery in ciliary ectosomes

Mammalian PAM is present in the intraluminal vesicles of MVBs and in extracellular vesicles purified from blood, saliva, and urine [32–35]. With a multilayered cell wall covering all but its ciliary membrane, the origin of the bioactive ectosomes released by vegetative cells and adhering gametes is clear [20,22,24,31].

Mass spectrometry of *C*. *reinhardtii* mating ectosomes identified 102 prepropeptides (including three amidated proteins) that fell into seven broad groups, as well as PAM, four subtilisin-like S8 domain–containing proteases, several MMPs, and 15 plasma membrane receptors. The amidated propeptides identified exhibit distinct developmental expression patterns. Although lacking a transmembrane domain, all three amidated propeptides were ectosome associated, suggesting a role for lipid anchors or covalent cross-links. The active sites of the subtilisin-like proteases identified in mating ectosomes should be exposed on the ectosomal surface. Thus, the proteases and amidating enzyme could generate peptidergic products on the ectosomal and/or ciliary membrane as well as within the secretory pathway lumen. Whether all processing steps occur within a single ectosome or the enzymes can function in *trans* (i.e., between two interacting membrane-bound compartments) remains to be determined.

GPCRs for a variety of peptides are targeted to mammalian cilia, where they participate in key developmental pathways and signal essential physiological responses, e.g. [52–54]. GPCRs have been identified in ectosomes released from IMCD3 kidney cells [55]. While *C*. *reinhardtii* cilia release ectosomes rapidly [20,22,24], IMCD3 cells bud a single ectosome every 1–2 hours, providing a mechanism to shut down receptor-mediated signaling by components that lack retrieval determinants and cannot be recycled to the cell body [55]. In *C*. *reinhardtii* ectosomes, we identified a single GPCR that most closely resembles human GPR107. GPR107 binds neuronostatin, a peptide produced from preprosomatostatin that stimulates glucagon release and attenuates glucose-stimulated insulin secretion [56]. *C*. *reinhardtii* mating ectosomes also contain several scavenger, lectin, TNF-like, and ionotropic glutamate receptors, as well as the blue light receptor—phototropin. Thus, *C*. *reinhardtii* ciliary ectosomes may both transmit and even possibly receive signals in a cell-autonomous manner.

### An ectosome-derived amidated peptide as a gamete chemotactic modulator

Chemotaxis of *C*. *reinhardtii* towards ammonium and bicarbonate is controlled by phototropin and a kinase cascade [41,57]. Here, we find that one identified amidated peptide derived from mating ectosomes (VLYPNDPAAYAAYAPGTGGGATI-amide) acts as a chemoattractant for minus gametes and as a chemo-repellent for plus gametes, suggesting a role for amidated products in chemokinesis. C-terminal amidation of the peptide was an essential determinant; neither the non-amidated peptide nor the glycine-extended precursor elicited a chemotactic response. To attract sperm, echinoderm eggs release small soluble amidated peptides that bind with high affinity to sperm tail receptors. Vespids, injected wasp venom peptides with amidated C-termini, induce chemotaxis in polymorphonuclear leukocytes and macrophages, enhancing the inflammatory effects of hornet stings [58,59]. The Cre03.g204500 bioactive amidated product identified here is part of a large protein bound to the ectosome surface; whether this larger amidated product exhibits similar chemotactic properties to the GATI-amide peptide is uncertain. As *C*. *reinhardtii* lives in soil (and see below), it seems likely that released ectosomes would often adsorb to soil particles, thereby leaving a track of exposed amidated products that might be detected by other cells and allowing for a chemotactic response in a mixed liquid/solid phase environment. Thus, use of amidated products to mediate chemotactic signaling is a highly conserved process found in chlorophyte algae and throughout the metazoa and thus likely dates to before the last eukaryotic common ancestor.

### Ectosome content is developmentally regulated

The release of CrPAM in ectosomes is developmentally regulated, with CrPAM protein and enzyme activity found in ectosomes from mating gametes but not in vegetative ectosomes or in the soluble mating secretome. The overall protein compositions of ectosomes from minus and plus vegetative cells and mating gametes are distinct, demonstrating that the cell exerts significant control over ectosome content (and see [22]). In vegetative cilia, CrPAM, a transmembrane protein, is not solubilized by detergent treatment, suggesting that it is tightly bound to the ciliary axoneme [12]; this association must be disrupted to allow CrPAM release in mating ectosomes. Polycystin 2 behaves in a somewhat similar manner [60].

The routes by which CrPAM and FMG1 traffic to cilia are different; FMG1, but not CrPAM, is found on the plasma membrane [12,61]. Immunocytochemical analyses of CrPAM and FMG1 released during the mating process revealed the presence of distinct subpopulations of ectosomes with varied protein cargoes. Furthermore, CrPAM ciliary puncta appeared periodic although not separated by a constant unitary length; whether this reflects dedicated axonemal docking sites or membrane-based exclusion zones is uncertain. Heterogeneity in both exosome and ectosome cargoes, where both luminal content and membrane composition differ, has been reported for several human cell types [62–64], suggesting broadly conserved mechanisms for the regulated release of proteins in extracellular vesicles.

### When might *C*. *reinhardtii* living in their natural environment use ectosomes for signaling?

The natural habitat of all confirmed *C*. *reinhardtii* isolates is nutrient-rich temperate soils, where they exist in the water layer covering soil particles [65–67]. Other *Chlamydomonas* species are found in soil crust, lakes, and ponds and both Antarctic and marine environments, making them a pioneer component of the biosphere [67,68]. In its natural habitat, *C*. *reinhardtii* encounters numerous and varied predators such as *Daphnia* [69], rotifers [70], large ciliates (e.g., *Tetrahymena* [71]), and euglenoids (e.g., *Peranema* [72]), as well as ever-changing, often harsh, environmental conditions. The fact that peptide amidation requires molecular oxygen and copper may directly link the production of signaling peptides to environmental conditions. Indeed, comparing CrPHM and CrPAL activities to CrPAM protein levels throughout the life cycle revealed independent regulation of CrPHM activity. This has not been observed for mammalian PHM and perhaps reflects formation of a disulfide bond linkage between the CrPHM and CrPAL domains through the unique Cys residues present in CrPHM and CrPAL, or the presence of PHM in a recently observed inactive, closed conformation [73].

*C*. *reinhardtii* form mutualistic relationships with various fungi [74] and disrupt bacterial quorum sensing using an unknown secreted factor [75]. To perform these activities, survive other biotic and abiotic challenges, and allow mating-competent gametes to find each other, *C*. *reinhardtii* must clearly engage in intercellular communication. For example, to avoid predation, individual *C*. *reinhardtii* cells join together to form aggregates too large to be engulfed [72]. Successful gamete interaction and mating leads to formation of zygospores that can remain viable under various biotic stress conditions [76–78]. Due to the presence of a multilayered cell wall, secretion from the plasma membrane does not lead to environmental release of proteins, and thus ectosome budding from the exposed ciliary membrane provides an alternate secretory pathway to enable cell–cell communication.

In conclusion, we describe here cilia-based peptidergic signaling through the release of ectosome-associated bioactive amidated products in the chlorophyte alga *C*. *reinhardtii*. Given that both the peptidergic signaling machinery and cilia have been highly conserved between algae and metazoans, we predict this process plays key roles in mammalian physiology, for example in brain, where primary cilia are embedded in a tissue and thus in direct contact with numerous surrounding cells.

## Materials and methods

### *C*. *reinhardtii* strains and growth conditions

Wild-type CC124 minus and CC125 plus mating type and fusion-deficient HAP2 minus *C*. *reinhardtii* strains were obtained from the *Chlamydomonas* Resource Center (https://www.chlamycollection.org/). Cells were cultured in R medium (Harris, 2009) aerated with 95% air and 5% CO_2_ under a 12-hour light/12-hour dark cycle at 22°C. To induce gametogenesis, vegetative cells were harvested from R medium, washed, and resuspended in nitrogen-deficient minimal medium (M-N medium) for 24–36 hours under a 12-hour light/12-hour dark cycle. Mating competency was determined by mixing an equal number of minus and plus mating type gametes and observing mating by light microscopy; only preparations in which >90% of gametes underwent the cilia-based agglutination reaction were used [13].

### Mixing of minus and plus gametes/zygote formation

Gametes of CC124 minus and CC125 plus mating type strains were prepared as described above. Equal numbers (5 × 10^6^ cells/mL) of gametes of each mating type were mixed for the indicated time; cells were harvested by centrifugation at 1,600*g* for 5 minutes and resuspended in 1X TMT buffer (20 mM 2-[tris(hydroxymethyl)-methylamino]-ethanesulfonic acid [TES], pH 7.4, 10 mM mannitol, 1% Triton X-100 Surfact-Amps, #28314, ThermoFisher Scientific, Waltham, MA) containing 0.2 M NaCl, a protease inhibitor cocktail (cOmplete ULTRA Tablets, Cat# 05892791001 Roche, Basel, Switzerland), and 0.3 mg/mL phenylmethylsulfonyl fluoride (PMSF). Unless otherwise noted, all standard chemicals were of highest available grade and were obtained from Sigma Chemical Co (St. Louis, MO). After two freeze–thaw rounds in microfuge tubes, cells were lysed using 3–4 pulses of sonication and centrifuged at 9,500*g* for 5 minutes to collect the soluble fraction (S) and insoluble fraction (P) of cell lysates. Insoluble fractions (P) were prepared in 1×SDS lysis buffer containing (0.5% [w/v] sodium dodecyl sulphate, 0.05 M Tris.Cl, pH 8.0, and 0.3 mg/mL PMSF). Samples were assayed for protein content using the bicinchoninic acid assay (BCA) (Thermo Fisher Scientific, Rockford, IL). Samples (30 μg) were prepared for SDS-PAGE by mixing with 2× Laemmli sample buffer (Bio-Rad, Hercules, CA) and denaturation at 55°C for 5 minutes. Criterion TGX 4%–15% polyacrylamide gradient gels (Bio-Rad, Hercules, CA) were used for fractionation and standard immunoblotting techniques and Coomassie blue staining were used after proteins were transferred to PVDF membranes. Immunoblots were quantified using Gene Tools software from Syngene (Frederick, MD). Rabbit Arf1 antibody was from Agrisera (cat# AS08 325; Vannas, Sweden).CrPHM and CrPAL enzyme activities in the soluble fractions (0.25 μg protein/sample) were measured as described, with 5 μM CuCl_2_ added for PHM assays [79].

### Immunofluorescence and image analysis

Minus and plus vegetative cells and gametes were harvested by centrifugation at 1,600*g*, resuspended in R/M-N medium, and fixed for 10 minutes at room temperature in 2% formalin in 30 mM HEPES, 5 mM EGTA, 5 mM MgSO_4_, 25 mM KCl, 4% sucrose, pH 7.0; cells were allowed to adhere to 0.1% polyethyleneimine-coated coverslips for 10 minutes and then treated with ice-cold methanol for 10 minutes at −20°C. Cells were washed with 1× phosphate buffered saline (PBS); permeabilized with PBS containing 0.5% Triton X-100 for 20 minutes at room temperature; and blocked with 3% fish skin gelatin (Sigma G7765), 1% bovine serum albumin (BSA), and 0.1% Tween-20 in PBS (blocking buffer) for 1 hour at room temperature. Cells were then incubated overnight at 4°C in primary antibodies prepared in blocking buffer; following washes, samples were then incubated for 1 hour at room temperature in secondary antibody diluted in blocking buffer. Primary antibodies used were affinity-purified rabbit CrPAM luminal domain antibody (1:500; CT319) , mouse FMG1 antibody (gift from Dr. R. Bloodgood, University of Virginia) and 6-11B-1 mouse α–Ac tub antibody (1:2,000; Ac tub) (Santa Cruz, Dallas, TX). Second antibodies used were Alexa Fluor 488–conjugated anti-rabbit IgG (1:1,000; Life Technologies #A11034; ThermoFisher Scientific, Rockford, IL) and Cy3-conjugated anti-mouse IgG (1:2,000; #715166151, Jackson Immuno Research Laboratories, West Grove, PA). Images were obtained using a Zeiss LSM 880 confocal microscope with 63×/1.4 Plan-Apochromat Oil objective. Maximum intensity projections of images are shown in Fig 2D. Image J software (https://imagej.nih.gov/ij/) was used to analyze CrPAM immunostaining. The number and intensity of PAM-positive ciliary puncta were analyzed with UNICORN 5.2 (GE Healthcare Life Sciences, Marlborough, MA).

### Preparation of ectosome-enriched pellets from mating gametes

Gametes were prepared from CC124 minus and CC125 plus vegetative cell as described above. Gametes of both mating types were washed and resuspended in 10 mL fresh M-N medium at a density of 8–10 × 10^6^ cells/mL. An equal number of mating type minus and plus gametes were mixed; after a 1-hour incubation, the cultures were centrifuged at 1,600*g* to pellet the cells. The supernatant was then centrifuged at 20,000*g* for 30 minutes at 4°C to pellet any cellular debris, resulting in a low-speed pellet (LSP). The 20,000*g* supernatant was centrifuged at 200,000*g* for 60 minutes at 4°C to sediment extracellular vesicles. The final supernatant is referred to as the soluble mating secretome (S) and the pellet is referred as the ectosome-rich pellet (E). The LSP and E were suspended in 1×TMT buffer containing 0.2 M NaCl, a protease inhibitor cocktail, and 0.3 mg/mL PMSF. Samples were assayed for protein content using the BCA assay. Mating ectosome samples were fractionated by SDS-PAGE, and proteins were visualized by silver staining (Silver Stain for Mass Spectrometry; Thermo Fisher Scientific, Rockford, IL).

For ectosome preparations from fusion defective HAP2 minus and wild-type CC125 plus gametes, gametes were treated with autolysin [80] for 30 minutes, washed, and resuspended in fresh M-N medium; ectosomes were prepared as described above.

### Preparation of vegetative ectosome-enriched pellets

Ectosomes were isolated from both minus and plus vegetative cells. Cells were grown for 5 days in 2 L of R medium, washed, and resuspended in 20 mL of fresh R medium (5–10 × 10^6^ cells/mL). After a 4-hour incubation under continuous light with gentle aeration, ectosome-rich pellets were prepared as described above. For immunoblotting and proteomic analyses, pellets were resuspended in 1× TMT containing 0.2 M NaCl, 0.3 mg/mL PMSF, and 1× protease inhibitor cocktail. Vegetative ectosomes were fractionated by SDS-PAGE and proteins were visualized by silver staining (Thermo Fisher Scientific, Rockford, IL).

### Electron microscopy

Mating ectosomes were resuspended in 10 mM HEPES buffer containing a protease inhibitor cocktail and 0.3 mg/mL PMSF. For negative staining, a 5-μL drop of the sample was placed onto a glow-discharged 400-mesh carbon-coated copper grid (Electron Microscopy Sciences, Hatfield, PA); after 30–60 seconds, excess liquid was removed. Uranyl acetate (1% w/v aq.) was applied to the grid for 30 seconds. Samples were imaged using a Hitachi H-7650 transmission electron microscope (Hitachi High Technologies Corporation, Tokyo, Japan) operating at 80 kV. For immunostaining, freshly isolated mating ectosomes were applied to grids as described above; after removal of excess sample, grids were incubated for 30 minutes at room temperature in blocking solution (1% BSA in PBS). Grids were then incubated in affinity-purified CrPAM luminal domain antibody (CT319; 1:10) in blocking solution for 1 hour at room temperature; after washing, grids were incubated in gold-conjugated goat antibody to rabbit immunoglobulin (10-nm gold; 1:15; Electron Microscopy Sciences, Hatfield, PA) in blocking solution for 1 hour at room temperature. Grids were then washed and stained with uranyl acetate (1% w/v aq.) for 30 seconds. Images were obtained as described above.

### Mass spectrometry

Six mating ectosome samples (each containing 2–5 μg protein) prepared from mixed CC124 minus/CC125 plus gametes at two different times were subjected to analysis, yielding Dataset 1 (Samples A, B, and C) and Dataset 2 (Samples D, E, and F). For mass spectrometry, electrophoresis was stopped after the dye band had traveled 3 cm and gels were stained with QC Colloidal Coomassie (Bio-Rad, Hercules, CA). Based on molecular weight standards analyzed at the same time, each lane was cut into 4 slices covering the entire molecular weight range. Gel slices were stored frozen before preparation for LC-MS/MS analysis after in-gel digestion with trypsin. Gel slices were cut into small pieces, washed with 600 μL H_2_O and then with 50% acetonitrile/100 mM NH_4_HCO_3_. After a final wash with 50% acetonitrile/25 mM NH_4_HCO_3_, samples were dried using a speed vacuum concentrator. Sample were suspended in 80 μL 4.5 mM dithiothreitol, 25 mM NH_4_HCO_3_, and incubated at 37°C for 30 minutes. Samples were alkylated by adding 80 μL 10 mM iodoacetamide, 25 mM NH_4_HCO_3_, and incubation at room temperature for 30 minutes in the dark. Samples were washed with 700 μL 50% acetonitrile/100 mM NH_4_HCO_3_ for 15 minutes on a tilt-table. Samples were washed a final time with 50% acetonitrile/25 mM NH_4_HCO_3_ for 15 minutes and dried using a speed vacuum concentrator. Each sample was suspended in 25 mM NH_4_HCO_3_ containing 0.20 μg digestion-grade trypsin (V5111, Promega, Madison, WI) and incubated at 37°C for 16 hours. The supernatant was acidified and placed into a vial for LC-MS/MS analysis (5 μL injected).

Reversed-phase LC-MS/MS was performed using a nanoACQUITY UPLC system (Waters Corporation, Milford, MA) connected to an Orbitrap Fusion Tribrid (ThermoFisher Scientific, San Jose, CA) mass spectrometer. After injection, samples were loaded into a trapping column (nanoACQUITY UPLC Symmetry C18 Trap column, 180 μm × 20 mm) at a flow rate of 5 μL/minutes and separated with a C18 column (nanoACQUITY column Peptide BEH C18, 75 μm × 250 mm). Mobile phases A and B were 0.1% formic acid in H_2_O and 0.1% formic acid in acetonitrile, respectively. Peptides were eluted with a gradient extending from 3% to 20% mobile phase B in 85 minutes and then to 35% mobile phase B in another 35 minutes at a flow rate of 300 nL/minute and a column temperature of 37°C. Data were acquired with the mass spectrometer operating in a top speed data-dependent mode. The full scan was performed in the range of 300–1,500 m/z at an Orbitrap resolution of 120,000 at 200 m/z and automatic gain control (AGC) target value of 4 × 10^5^. The full scan was followed by MS2 event of the most intense ions above an intensity threshold of 5 × 10^4^. The ions were iteratively isolated with a 1.6 Th window, injected with a maximum injection time of 110 ms, AGC target of 1 × 10^5^, and fragmented with higher-energy collisional dissociation.

### Mass spectrometry data analysis

Raw data were processed using Proteome Discoverer software (Version 2.1, ThermoFisher Scientific, San Jose, CA). MS2 spectra were searched using Mascot (Matrix Science, London, United Kingdom) and Byonic (Protein Metrics, San Carlos, CA) set up to search against the *C*. *reinhardtii* database (Creinhardtii_281_v5.5). The Mascot search criteria included 10 ppm precursor mass tolerance, 0.02-Da fragment mass tolerance, trypsin enzyme, and maximum missed cleavage sites of two. The Byonic search criteria included 10 ppm precursor mass tolerance, 20 ppm fragment mass tolerance, trypsin enzyme, and maximum missed cleavage sites of three. Dynamic modifications included propionamide on cysteine, oxidation on methionine, deamidation on asparagine and glutamine, Gly-loss+Amide on C-terminal glycine, and carbamidomethylation on cysteine (Byonic search). Peptide spectral match (PSM) error rates were determined using the target-decoy strategy coupled to Percolator modeling of true and false matches. Scaffold (version 4.8.4, Proteome Software, Portland, OR) was used to validate MS/MS-based peptide and protein identifications. Peptide identifications were accepted if they could be established at greater than 95.0% probability by the Scaffold local FDR algorithm. Protein identifications were accepted if they could be established at greater than 99% probability and contained at least 2 identified peptides. Proteins that contained similar peptides and could not be differentiated based on MS/MS analysis alone were grouped to satisfy the principles of parsimony. Proteins sharing significant peptide evidence were grouped into clusters.

### Bioinformatic analyses

A total of 2,715 proteins were identified in combined Datasets 1 and 2. Proteins recognized in fewer than four of the six samples were eliminated, yielding a Merged Dataset of 1,889 proteins (S1 Table). We used PredAlgo (https://giavap-genomes.ibpc.fr/predalgo/) and Signal P 4.1 (www.cbs.dtu.dk/services/SignalP/) to predict signal peptide (SP)–containing proteins and the TMHMM Server v. 2.0 (http://www.cbs.dtu.dk/services/TMHMM/) to predict TMH-containing proteins. The merged dataset contained 247 signal peptide–containing proteins (S3 Table) and 38 proteins with TMHs (S5 Table). Multiple sequence alignments and phylogenetic trees of prepropeptide precursors identified in mating ectosome (Figs 4A and S4 and S7 Table) were made using ClustalW (https://www.genome.jp/tools-bin/clustalw) and the interactive tree of life tool (iTOL) (https://itol.embl.de/). Gene annotation screening identified 15 plasma membrane receptor proteins (S1 Table). A list of 33 identified proteases is given in S8 Table. Proteins in mating ectosomes were grouped on the basis of function as assigned by Phytozome v12.1 (https://phytozome.jgi.doe.gov) and literature searches. The Gly-loss+Amide screen identified three amidated peptides in all six samples (Figs 5 and S3). Sequences homologous to the amidated proteins were aligned using the multiple sequence alignment program T-Coffee (http://tcoffee.crg.cat/). NeuroPred (http://stagbeetle.animal.uiuc.edu/cgi-bin/neuropred.py) and SMART (http://smart.embl-heidelberg.de/) were used to predict cleavage sites and protein domains.

### Chemotaxis assays

Petri dishes contained 1.5% agar in M-N medium. Peptides (1 mM stock) were diluted and mixed with 500 μL of 1% low melting point agarose (Catalog # A9539-50G Sigma, St. Louis, MO) to make 10 μM peptide-containing agarose blocks. Equal volumes (500 μL) of agarose in M-N medium alone or M-N medium containing 0.1% DMSO (dimethyl sulfoxide, Catalog # D5879 Sigma, St. Louis, MO) were used to make control blocks. CC124 minus or CC125 plus gametes were uniformly spread on the surface of the agar. Agarose blocks containing 10 μM GATI-amide, 10 μM GATI-OH, M-N medium, or M-N medium containing 0.1% DMSO were placed onto the Petri dish and the Petri dish was wrapped with aluminum foil to avoid any phototactic effects.

Microfluidic channel slides (μ slide I^0.2^ Luer; ibidi GmbH, Gräfelfing, Germany) were used for gradient formation. Two chambers, one serving as the cell reservoir and other as a chemo-effector reservoir, are connected by a microfluidic channel with dimensions of 50 mm × 5 mm × 0.2 mm. Channel volume is 50 μL and reservoir volume is 60 μL. Microfluidic channel slides were placed into Petri dishes humidified with M-N medium–soaked paper towels. To set up the gradient, the microfluidic channel was filled with 50 μL M-N medium; 10 μL of peptide/control medium was added to the chemo-effector reservoir and 10 μL was removed from the cell reservoir. Slides were kept at room temperature for 15 minutes to allow a stable gradient to form (Application Note 01, https://ibidi.com/). Gametic cells (10 μL, 1.2 × 10^6^ cells/mL) were added to the cell reservoir chamber in the dark. Images were obtained at the specified times using an inverted microscope (Nikon Eclipse TE300; Nikon Instruments, Melville, NY) with a 4× objective lens. A red filter was used throughout the imaging process to avoid any phototactic effects. The whole experiment was conducted in a darkened room. The number of cells in each image was counted using the mosaic plugin in Image J (http://rsb.info.nih.gov/ij/) and manually. The manual tracking plugin (http://rsb.info.nih.gov/ij/plugins/track/track.html) was used to track the cells. The data were analyzed using the chemotaxis and migration tool software (https://ibidi.com/). To characterize gradient formation and stability, a gradient was generated using 1 μM FITC-tagged peptide (FITC-Ahx-GPGDFSTYV; molecular mass, 1542 Da). Fluorescent images of the entire channel length were obtained at the indicated times. The mean gray value of each image was obtained using Image J software. Fluorescence intensity was calculated along the channel and is plotted for each time point (S5 Fig).

### Quantification and statistical analysis

For each experiment, the number of biological replicates appears in the figure legend. One-way ANOVAs with Tukey’s multiple comparison test were used to compare the means. Results are represented as mean ± SEM or as indicated. For statistical analysis of the CI in Fig 6, one-way ANOVAs with Tukey’s multiple comparison test and two-way ANOVAs with Bonferroni posttests were used. GraphPad prism 5 software was used to perform all statistical analyses. Chemotaxis and migration tool software were used to generate the trajectory plots, calculate the COM, and perform the Rayleigh endpoint test for uniform circular point distribution and calculate *P* values; *P* < 0.05 rejects uniformity.

## Supporting information

S1 Fig Transcriptomic changes in gene expression during the sexual life cycle of *Chlamydomonas* (data from Ning and colleagues, 2013). Transcript levels were reported for asynchronous vegetative cells and for vegetative cells synchronized using a light/dark cycle, minus and plus resting gametes, and gametes activated with lysin (to remove the cell wall) or with db-cAMP (to mimic flagellar activation of the mating signaling pathway) [29]. Cre03.g204500 expression is up-regulated in resting gametes, rising further in db-cAMP activated gametes. Cre12.g487700 is more highly expressed in vegetative cells and resting gametes, dropping substantially after lysin and db-cAMP treatment. In contrast, Cre17.g722300 expression is down-regulated following lysin and db-cAMP treatment. The underlying data for this figure can be found in S1 Data. db, dibutyryl.
(TIF)

S2 Fig PAM localization in minus and plus vegetative cells and gametes. **A.** Histogram showing the percent of cilia on minus and plus vegetative and gametic cells having the indicated number of PAM-positive puncta; 30–70 cilia were analyzed for each group. **B.** Kolmogorov-Smirnov plot showing the cumulative distribution of PAM-positive puncta of increasing intensity in vegetative cells and gametes of both mating types showed no statistically significant differences. These quantitative data support the stained cell images shown in Fig 2D. The underlying data for this figure can be found in S1 Data. PAM, peptidylglycine α-amidating monooxygenase.
(TIF)

S3 Fig Analysis of ectosome-rich pellets and identification of amidated products. **A.** The amounts of protein recovered in the ectosome-rich pellets compared to the initial cell pellets were quantified for three samples of minus and plus vegetative ectosomes and for six mating ectosome samples. Ectosomal protein production by minus and plus vegetative cells (over a 4-hour period) and by mating gametes (over a 1-hour period) was expressed as a percentage of total cell protein ± SEM. The underlying data for this figure can be found in S1 Data. **B.** Predalgo and SignalP analyses were performed to identify signal peptide–containing proteins. Functional predictions were made using Phytozome and literature analyses. **C.** MS/MS fragmentation spectra of amidated peptides. Data for the amidated peptides identified are shown: Cre03.g204500 (VLYPNDPAAYAAYAPGTGGGATI-amide), Cre12.g487700 (PLVPAAA-amide), and Cre17.g722300 (GELNPAGGQLPG-amide). Amidated peptide identification was carried out using Byonics software, which assigns a score (0 to 1,000) that serves as an indicator of peptide spectrum match correctness. Assigned scores for the spectra shown are indicated. As can be seen for the amidated peptide derived from Cre17.g722300, multiple b- (red) and y- (blue) fragment ions were observed; y-ions y3, y4, y7, y9, and y10 showed loss of a glycine and the presence of an amidated C terminus (GELNPAGGQLPG-amide vs GELNPAGGQLPGG). MS, mass spectrometry.
(TIF)

S4 Fig Cre03.g204500 is part of a large gene family. **A.** Neighbor joining rooted phylogenetic tree of sequences related to Cre03.g204500 (red arrow) that returned BLASTP E value scores of 4 × 10^−20^ or less. Although ectosome-associated Cre02.g102050 exhibits 31% identity to and clusters with Cre03.g204500 (Fig 3B), much of the conservation is in low-complexity regions, resulting in a BLASTP score considerably below the cutoff used here. **B.** Map of the Chromosome 8 genomic region that contains 11 clustered closely related genes that show considerable similarity (up to approximately 40% identity) with the C-terminal region of Cre03.g204500. **C.** CLUSTALW sequence alignment of Cre03.g204500 and one member of the Chromosome 8 gene cluster (Cre08.g365100) revealed considerable sequence identity with the Cre03.g204500 C-terminal region, including 8 identical residues at the N terminus of the GATI-amide peptide (blue box). GATI-amide, VLYPNDPAAYAAYAPGTGGGATI-amide.
(TIF)

S5 Fig Profile of microfluidic gradient over time. The gradient was generated using a 1 μM stock of FITC-tagged peptide (FITC-Ahx-GPGDFSRYV) with a molecular weight similar to that of GATI-amide (2,209.46 Da). The gradient was generated as described in Materials and methods, and fluorescent images of the entire channel were obtained at the indicated times. The mean fluorescence intensity across the channel is plotted for each time point. The gradient was quite stable from 1 to 6 hours, flattening out somewhat after overnight incubation. The underlying data for this figure can be found in S1 Data. FITC, fluorescein isothiocyanate; GATI-amide, VLYPNDPAAYAAYAPGTGGGATI-amide.
(TIF)

S6 Fig Analysis of CrPAM and FMG1 localization at different times after mixing minus and plus gametes. CC124− and CC125+ gametes were fixed at the indicated times after mixing, permeabilized, and stained for FMG1 (red) and for the luminal domain of CrPAM (green). Images shown are maximal projection images, except for the inset in the top left panel, which is a single plane of FMG1 staining at 0 minutes; a differential interference contrast image of each cell is also shown. Images are representative of three independent experiments. Scale bars = 5 μm. CrPAM, *C*. *reinhardtii* peptidylglycine α-amidating monooxygenase; FMG1, flagellar membrane glycoprotein 1.
(TIF)

S1 Table Annotated receptors encoded by the *C*. *reinhardtii* genome. **All receptors:** A gene annotation screen identified 146 receptors in the *C*. *reinhardtii* genome. Receptors were assigned to 12 groups based on their putative structure and function. Accession number and Phytozome annotation are provided; the number of receptors in each group is indicated in the row identifying that group. **Receptors in mating ectosomes:** The 15 annotated receptors identified in mating ectosomes are listed. **Expression analysis:** Published transcriptomic datasets from [29] and [30] were used to assess developmentally regulated expression of these receptors.
(XLSX)

S2 Table Proteins identified in mating ectosome-rich pellets. Total spectral counts for each sample were normalized to the average value. Normalized total spectral counts (Mean) from Datasets 1 (Samples A, B, C) and 2 (Sample D, E, F) and relative standard deviation are given. Accession numbers, Phytozome annotation, molecular mass, and Pred Algo predictions are tabulated. C, chloroplast; M, mitochondrion; NA, not annotated; O, other (cytosolic, vacuolar, endosomal); SP, signal peptide.
(XLSX)

S3 Table Signal peptide containing proteins. The signal peptide containing proteins identified in mating ectosomes were grouped based on function: cell wall proteins (23%); metabolic enzymes (19%), ER/Golgi-related proteins (8%), flagellar related proteins (4%); other (includes scavenger receptor cysteine-rich [SCRC] proteins, TRP channels, copper transport proteins, ionotropic glutamate receptors, ABC transporters, and proteins involved in RNA biogenesis and translation). Accession number, Phytozome annotation, molecular mass, normalized total spectral counts (mean), and relative standard deviation are shown. ABC, ATP-binding cassette; ER, endoplasmic reticulum; TRP, transient receptor potential.
(XLSX)

S4 Table The most prevalent signal peptide–containing proteins. Accession number, Phytozome annotation, molecular mass, normalized total spectral counts (mean), and relative standard deviation are shown. Six cell wall–related proteins, four flagellar-associated proteins, and FMG1 fall into this group. Three MMPs (MMP3, MMP13, G-lysin) were included. Four enzymes known to play a role in protein synthesis (aromatic amino acid transaminase, translation initiation factor 4A, Glu-tRNA synthetase, and Ala-tRNA ligase), and previously identified in exosomes [81, 82] were identified. FMG1, flagellar membrane glycoprotein 1; MMP, matrix metalloproteinase.
(XLSX)

S5 Table Membrane proteins in mating ectosomes. To explore proteins identified in mating ectosomes but placed into the Other (O) category based on Pred Algo analysis (S1 Table), TMHMM analysis was used to search for transmembrane domains (http://www.cbs.dtu.dk/services/TMHMM/); 38 additional proteins in mating ectosomes were identified as membrane proteins. Accession number, number of predicted transmembrane helices (#TMHs), and Phytozome annotation are provided.
(XLSX)

S6 Table Comparison of signal peptide–containing proteins in mating ectosomes and the soluble mating secretome. A comparison of signal peptide–containing proteins identified in the soluble mating secretome [13] and mating ectosomes (present study) yielded a list of 72 proteins common to both datasets, 176 proteins unique to mating ectosomes, and 27 proteins unique to the soluble mating secretome.
(XLSX)

S7 Table Predicted *C*. *reinhardtii* prepropeptides found in mating ectosomes. Our previous analysis identified 771 proteins encoded by the *C*. *reinhardtii* genome that have the characteristics expected of prepropeptides [13]. Ninety-nine of these proteins were identified in mating ectosomes. Accession number, Phytozome annotation, and gene symbol (when assigned) are provided, along with normalized total spectral counts, relative standard deviation, and information on the presence and number of putative prohormone convertase cleavage sites, amidation sites, furin cleavage sites, and furin/amidation sites.
(XLSX)

S8 Table Proteases identified in mating ectosomes. **Proteases:** Proteases were grouped based on their catalytic domains and the MEROPS database. Accession number, Phytozome annotation, assigned name, Pred Algo, TMHMM prediction, molecular mass, normalized total spectral counts, and relative standard deviation are reported, along with a brief description, the closest human homologue, and the MEROPS ID, clan, and family/Uniprot description. Gray highlighting identifies mating ectosomal proteases that were also in the soluble mating secretome [13]. **Expression analysis:** Published transcriptomic datasets [29, 30] were used to assess developmentally regulated expression of transcripts encoding the proteases identified in mating ectosomes.
(XLSX)

S1 Raw Images Raw annotated immunoblot and electrophoretic gel images for Figs 2A, 3A, 3B, 3F, 7A–7D and 8C. (PDF)

S1 Data Numeric data underlying the graphical plots shown in Figs 2B, 2C, 2E, 2F, 3C, 3E, 4B, 5B, 5C, 6C–6E, 6G, 6H, 7A–7E, 8B and 8D and S1, S2A, S2B, S3A and S5. (XLSX)

## Abbreviations

Ac tub: acetylated tubulin
AGC: automatic gain control
ARF1: ADP ribosylation factor 1
BCA: bicinchoninic acid assay
BSA: bovine serum albumin
CI: chemotaxis index
COM: center of mass
db: dibutyryl
ECM: extracellular matrix
ER: endoplasmic reticulum
FITC: fluorescein isothiocyanate
FMG1: flagellar membrane glycoprotein 1
FPKM: fragments per kilobase of transcript per million mapped reads
GATI-amide: VLYPNDPAAYAAYAPGTGGGATI-amide
GnRH: gonadotropin hormone releasing hormone
GPCR: G protein–coupled receptor
LSP: low-speed pellet
MMP: matrix metalloproteinase
M-N medium: gametic medium
MVB: multivesicular body
PAL: peptidyl-α-hydroxyglycine α-amidating lyase
PAM: peptidylglycine α-amidating monooxygenase
PBS: phosphate buffered saline
PCSK7: prohormone convertase PC7
PHM: peptidylglycine α-hydroxylating monooxygenase
PMSF: phenylmethylsulfonyl fluoride
PSM: peptide spectral match
TES: 2-[tris(hydroxymethyl)-methylamino]-ethanesulfonic acid
TMH: transmembrane helix
TNF: tumor necrosis factor
UPGMA: unweighted paired group method with arithmetic mean
VLE1: vegetative lytic enzyme 1
