# Supplementary figures and images for: Cilia-based peptidergic signaling
Source: PLoS Biol. 2019 Dec 6;17(12):e3000566. doi: 10.1371/journal.pbio.3000566 (PMC6919629; doi:10.1371/journal.pbio.3000566)

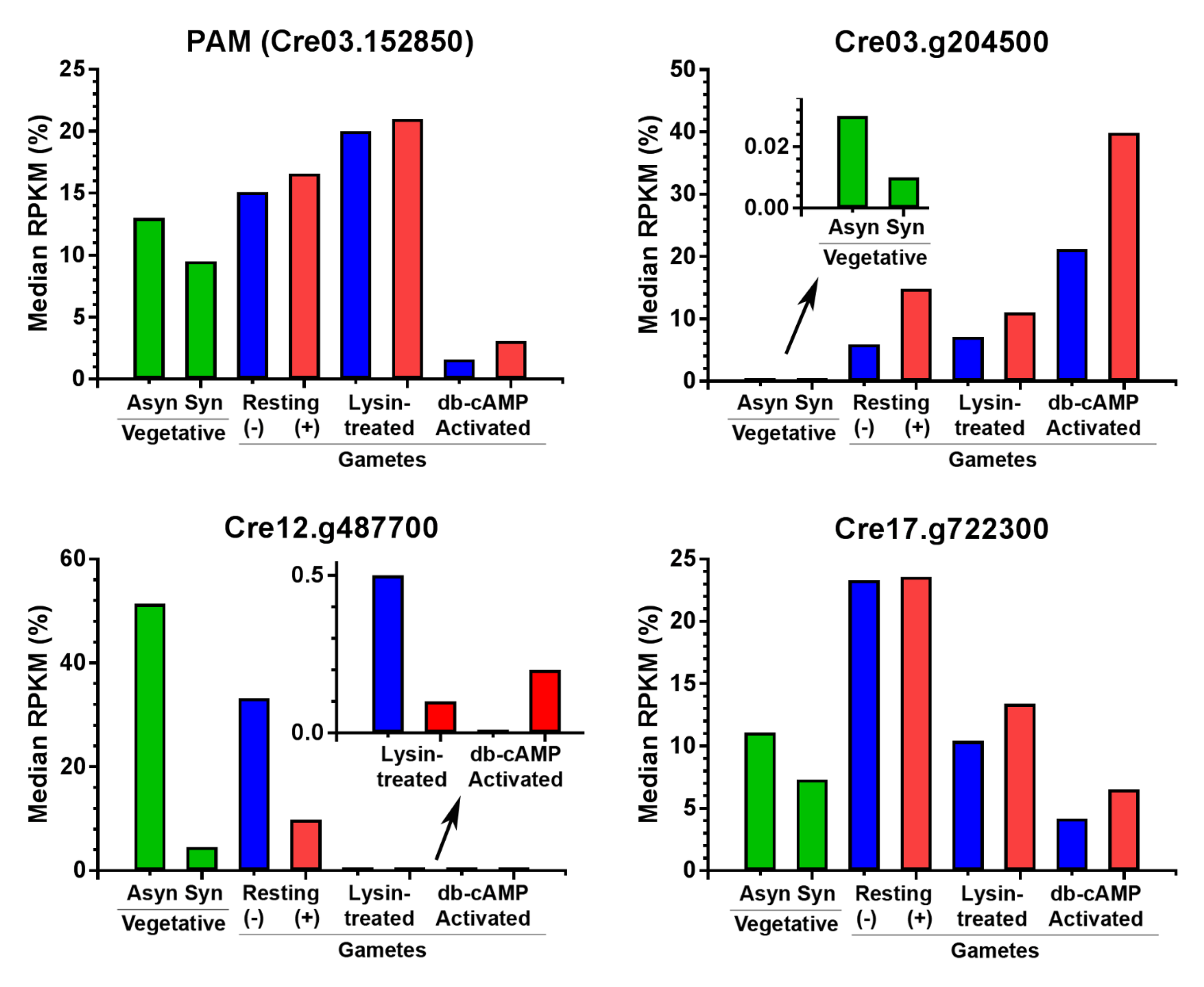

Supplement: S1 Fig — Transcript levels were reported for asynchronous vegetative cells and for vegetative cells synchronized using a light/dark cycle, minus and plus resting gametes, and gametes activated with lysin (to remove the cell wall) or with db-cAMP (to mimic flagellar activation of the mating signaling pathway) [29]. Cre03.g204500 expression is up-regulated in resting gametes, rising further in db-cAMP activated gametes. Cre12.g487700 is more highly expressed in vegetative cells and resting gametes, dropping substantially after lysin and db-cAMP treatment. In contrast, Cre17.g722300 expression is down-regulated following lysin and db-cAMP treatment. The underlying data for this figure can be found in S1 Data. db, dibutyryl. (TIF) [file pbio.3000566.s001.tif]

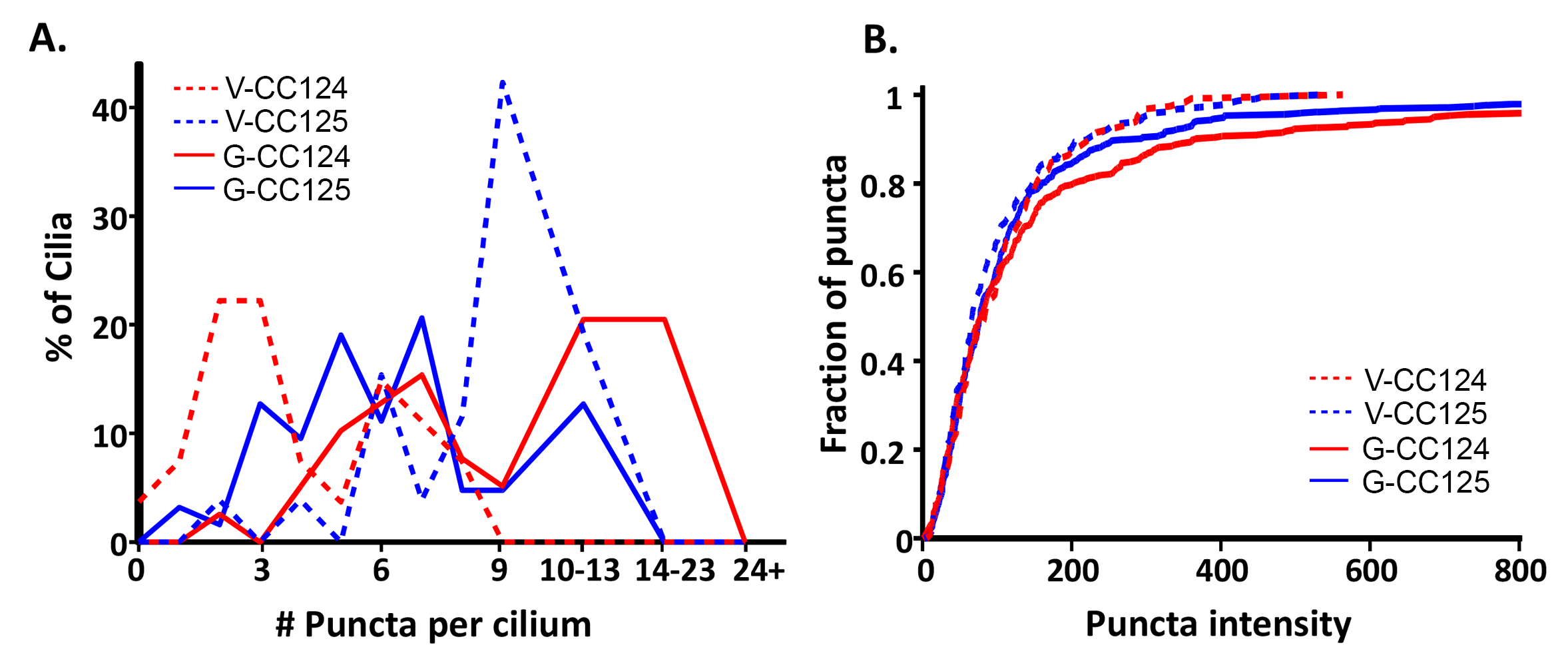

Supplement: S2 Fig — A. Histogram showing the percent of cilia on minus and plus vegetative and gametic cells having the indicated number of PAM-positive puncta; 30–70 cilia were analyzed for each group. B. Kolmogorov-Smirnov plot showing the cumulative distribution of PAM-positive puncta of increasing intensity in vegetative cells and gametes of both mating types showed no statistically significant differences. These quantitative data support the stained cell images shown in Fig 2D. The underlying data for this figure can be found in S1 Data. PAM, peptidylglycine α-amidating monooxygenase. (TIF) [file pbio.3000566.s002.tif]

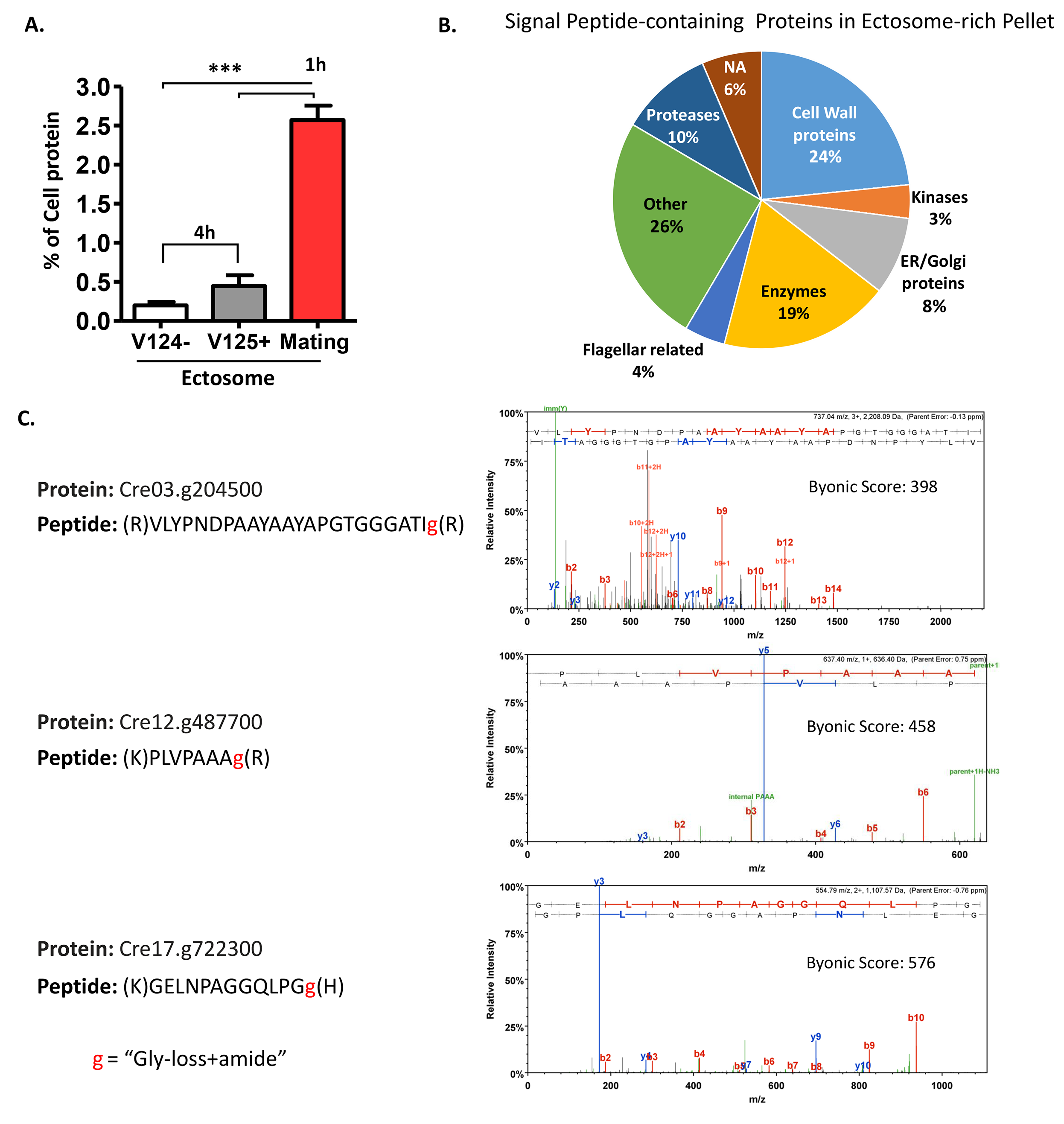

Supplement: S3 Fig — A. The amounts of protein recovered in the ectosome-rich pellets compared to the initial cell pellets were quantified for three samples of minus and plus vegetative ectosomes and for six mating ectosome samples. Ectosomal protein production by minus and plus vegetative cells (over a 4-hour period) and by mating gametes (over a 1-hour period) was expressed as a percentage of total cell protein ± SEM. The underlying data for this figure can be found in S1 Data. B. Predalgo and SignalP analyses were performed to identify signal peptide–containing proteins. Functional predictions were made using Phytozome and literature analyses. C. MS/MS fragmentation spectra of amidated peptides. Data for the amidated peptides identified are shown: Cre03.g204500 (VLYPNDPAAYAAYAPGTGGGATI-amide), Cre12.g487700 (PLVPAAA-amide), and Cre17.g722300 (GELNPAGGQLPG-amide). Amidated peptide identification was carried out using Byonics software, which assigns a score (0 to 1,000) that serves as an indicator of peptide spectrum match correctness. Assigned scores for the spectra shown are indicated. As can be seen for the amidated peptide derived from Cre17.g722300, multiple b- (red) and y- (blue) fragment ions were observed; y-ions y3, y4, y7, y9, and y10 showed loss of a glycine and the presence of an amidated C terminus (GELNPAGGQLPG-amide vs GELNPAGGQLPGG). MS, mass spectrometry. (TIF) [file pbio.3000566.s003.tif]

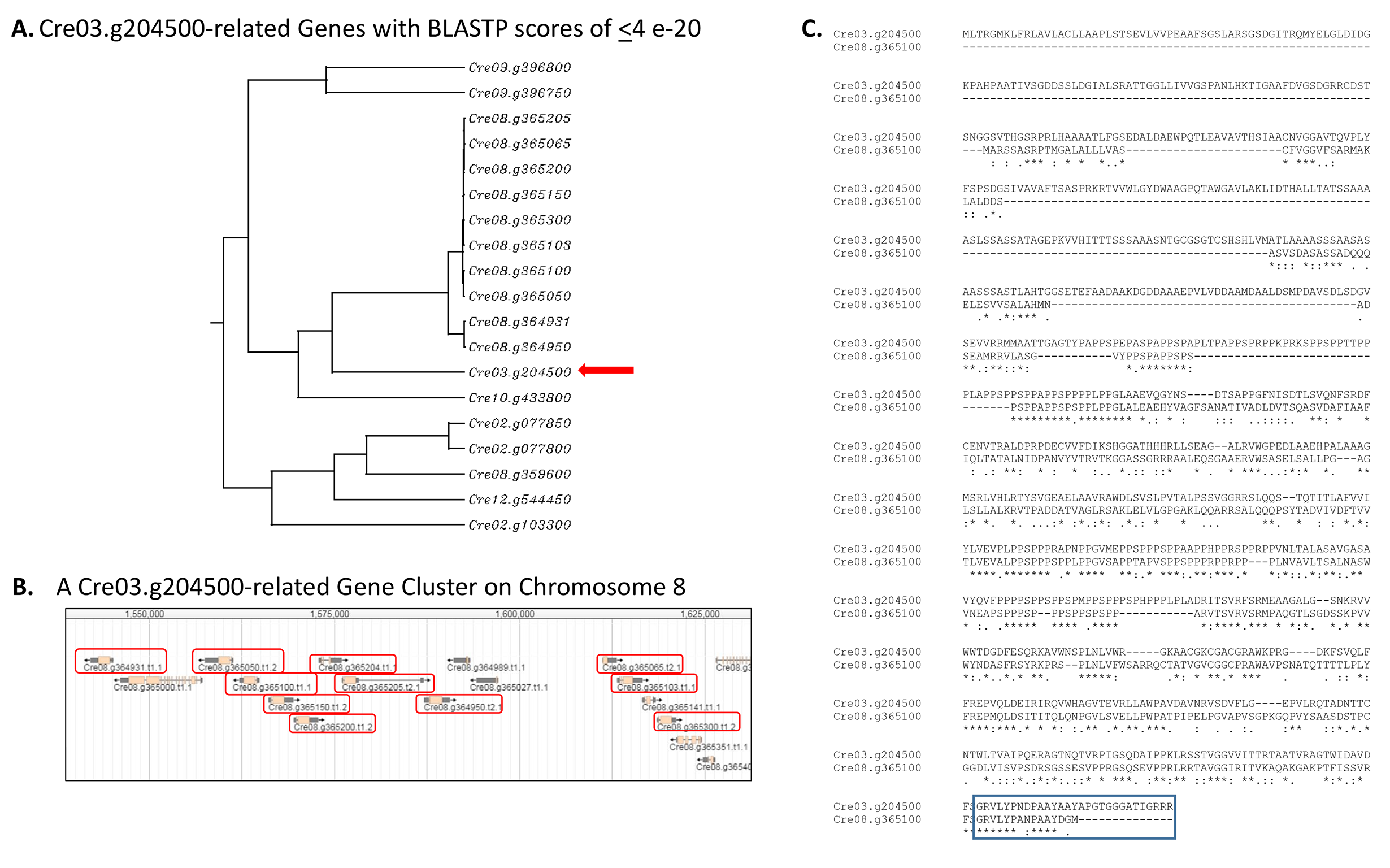

Supplement: S4 Fig — A. Neighbor joining rooted phylogenetic tree of sequences related to Cre03.g204500 (red arrow) that returned BLASTP E value scores of 4 × 10^−20^ or less. Although ectosome-associated Cre02.g102050 exhibits 31% identity to and clusters with Cre03.g204500 (Fig 3B), much of the conservation is in low-complexity regions, resulting in a BLASTP score considerably below the cutoff used here. B. Map of the Chromosome 8 genomic region that contains 11 clustered closely related genes that show considerable similarity (up to approximately 40% identity) with the C-terminal region of Cre03.g204500. C. CLUSTALW sequence alignment of Cre03.g204500 and one member of the Chromosome 8 gene cluster (Cre08.g365100) revealed considerable sequence identity with the Cre03.g204500 C-terminal region, including 8 identical residues at the N terminus of the GATI-amide peptide (blue box). GATI-amide, VLYPNDPAAYAAYAPGTGGGATI-amide. (TIF) [file pbio.3000566.s004.tif]

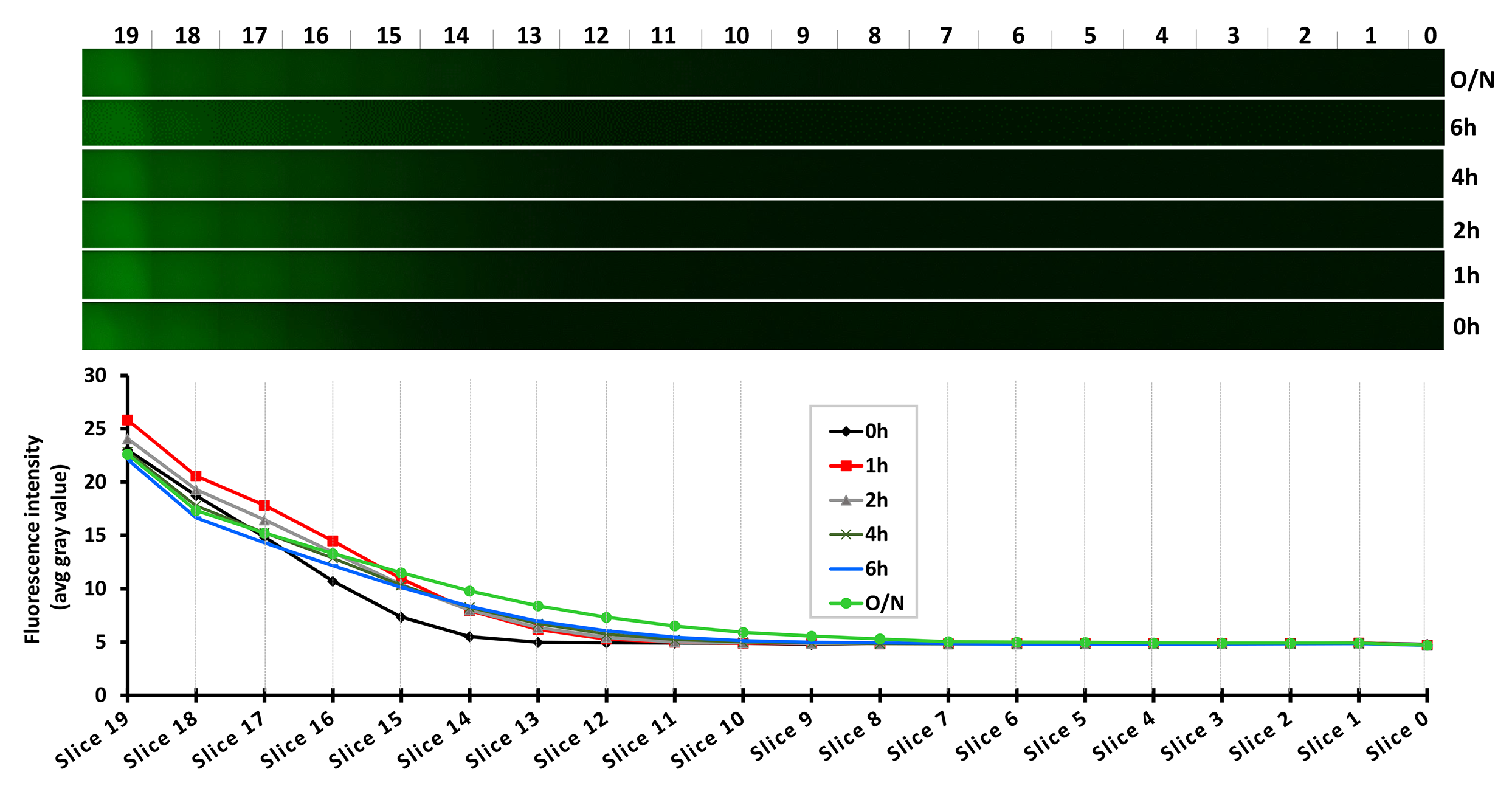

Supplement: S5 Fig — The gradient was generated using a 1 μM stock of FITC-tagged peptide (FITC-Ahx-GPGDFSRYV) with a molecular weight similar to that of GATI-amide (2,209.46 Da). The gradient was generated as described in Materials and methods, and fluorescent images of the entire channel were obtained at the indicated times. The mean fluorescence intensity across the channel is plotted for each time point. The gradient was quite stable from 1 to 6 hours, flattening out somewhat after overnight incubation. The underlying data for this figure can be found in S1 Data. FITC, fluorescein isothiocyanate; GATI-amide, VLYPNDPAAYAAYAPGTGGGATI-amide. (TIF) [file pbio.3000566.s005.tif]

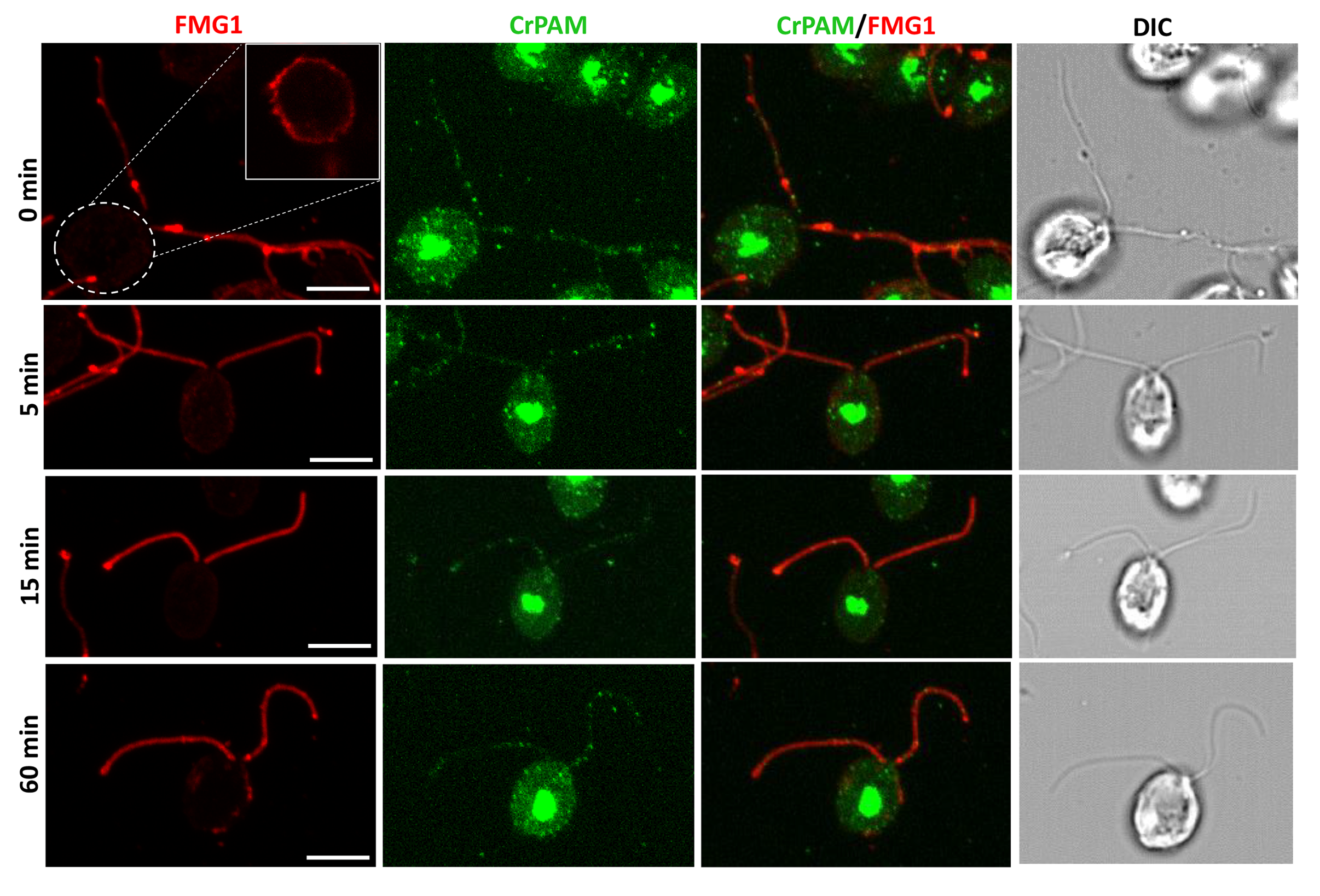

Supplement: S6 Fig — CC124− and CC125+ gametes were fixed at the indicated times after mixing, permeabilized, and stained for FMG1 (red) and for the luminal domain of CrPAM (green). Images shown are maximal projection images, except for the inset in the top left panel, which is a single plane of FMG1 staining at 0 minutes; a differential interference contrast image of each cell is also shown. Images are representative of three independent experiments. Scale bars = 5 μm. CrPAM, C. reinhardtii peptidylglycine α-amidating monooxygenase; FMG1, flagellar membrane glycoprotein 1. (TIF) [file pbio.3000566.s006.tif]
