# Supplementary material for: Cilia-based peptidergic signaling
Source: PLoS Biol. 2019 Dec 6;17(12):e3000566. doi: 10.1371/journal.pbio.3000566 (PMC6919629; doi:10.1371/journal.pbio.3000566)

Fig. 2A_coomassie stained_raw image

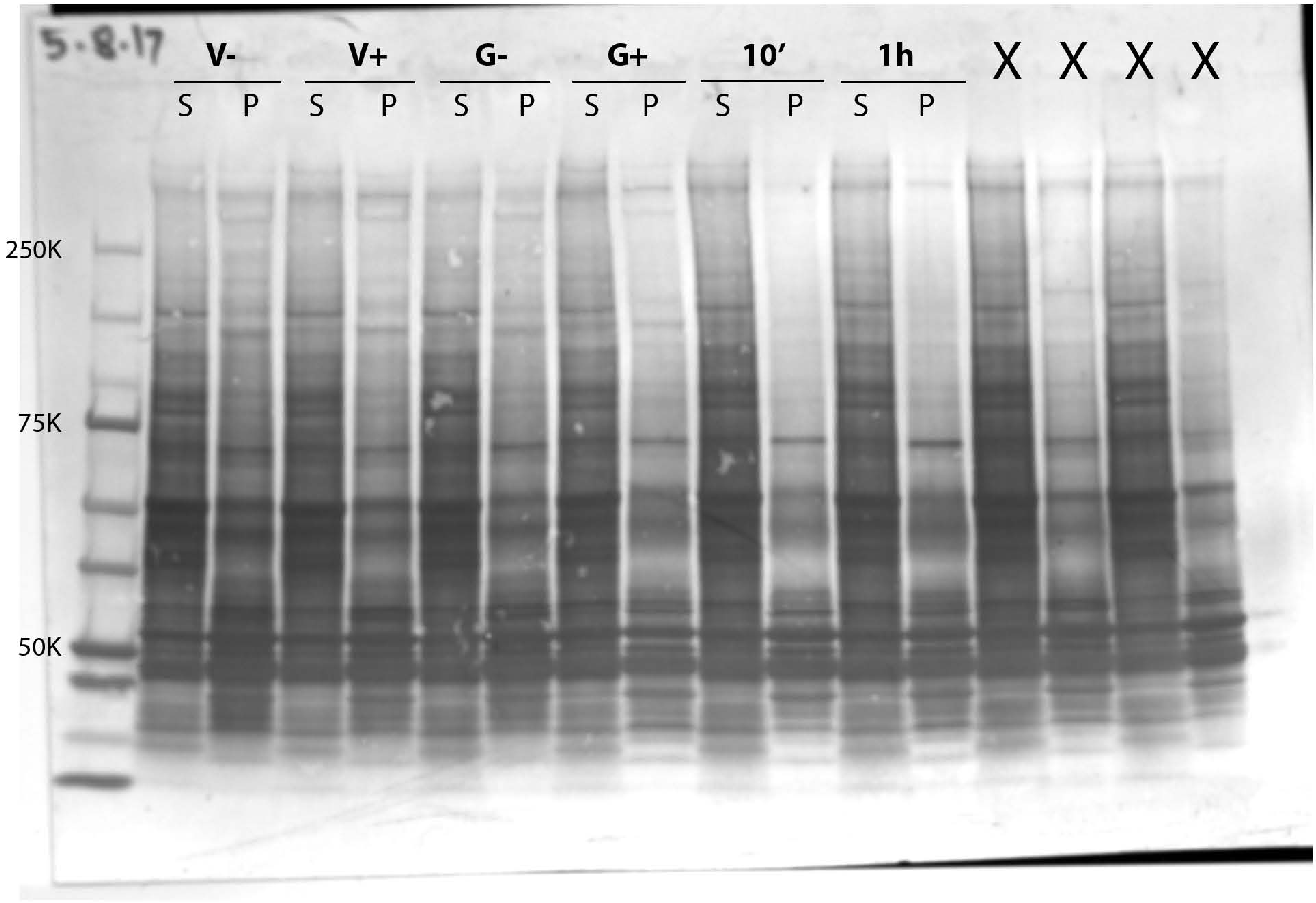

Fig. 2A_raw image

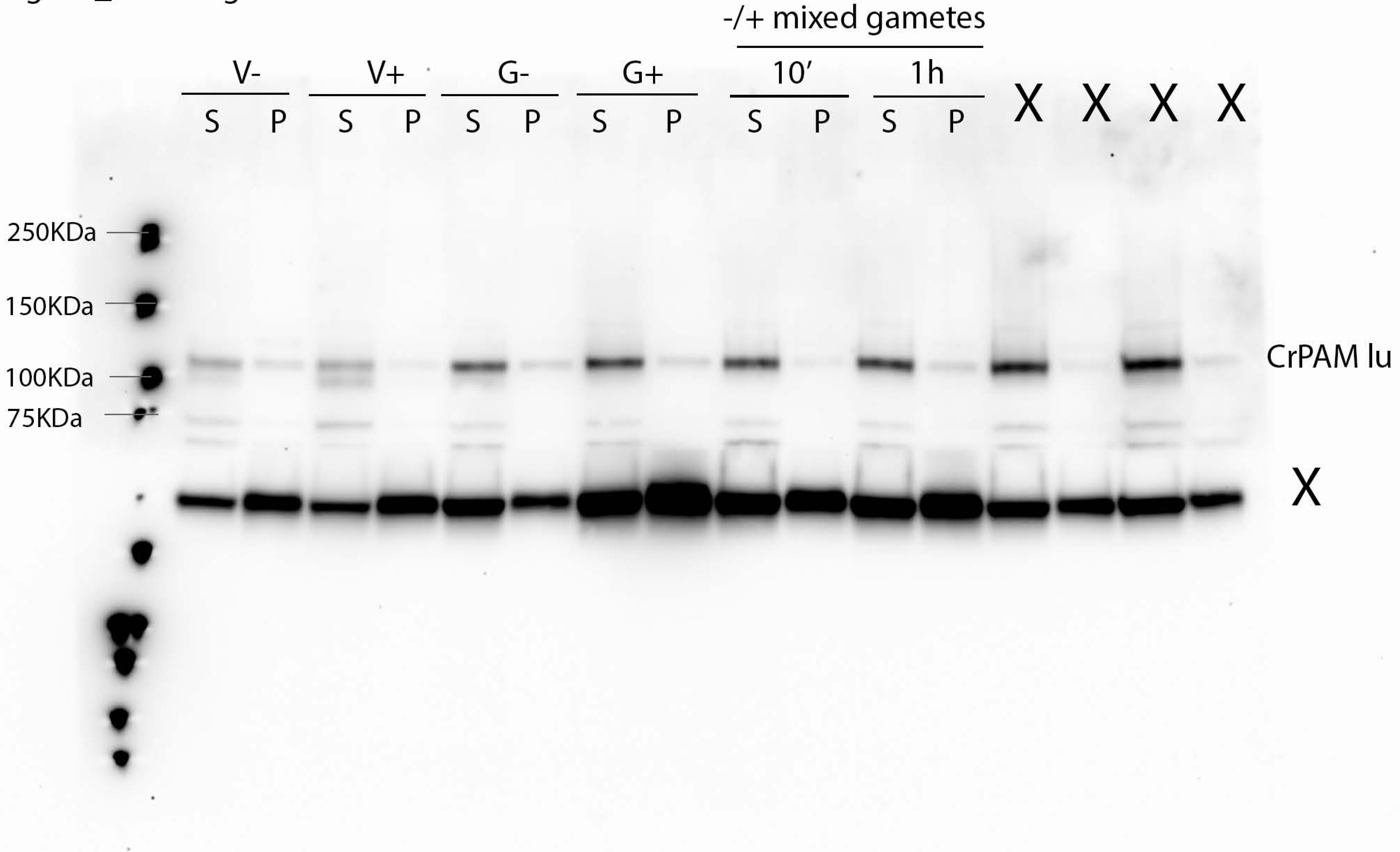

Fig. 3A_raw image

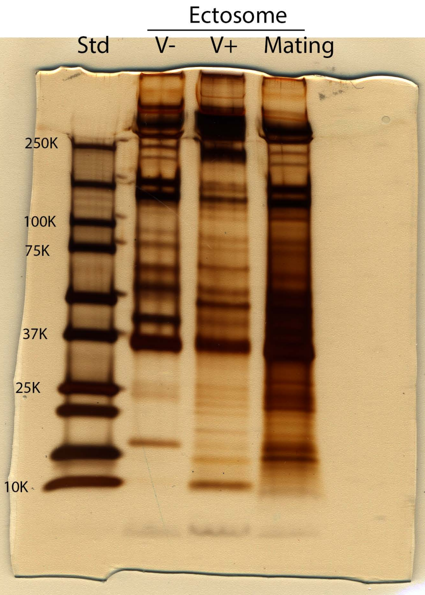

Fig. 3B_raw_image

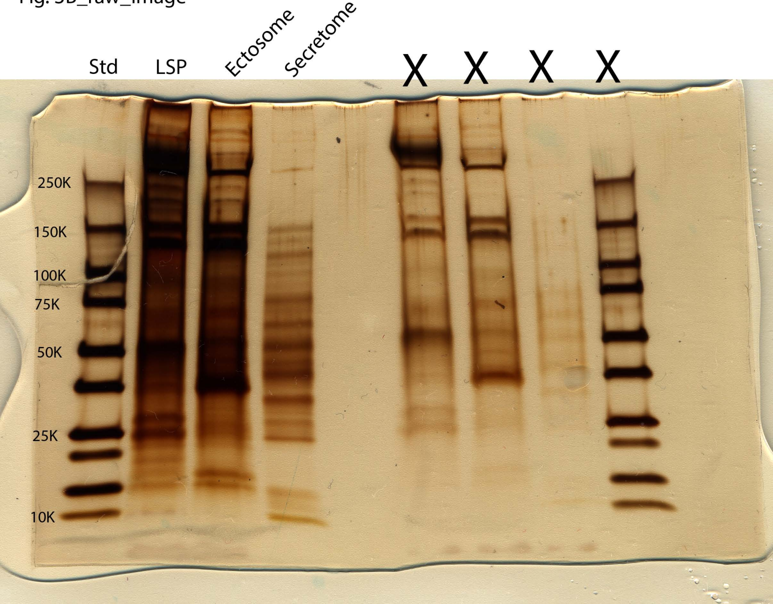

Fig. 3F_raw_image

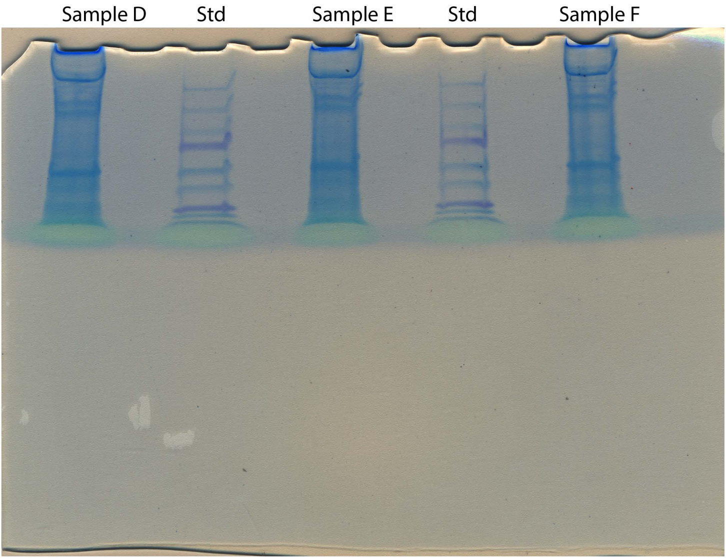

Sample D, E, F - Mating ectosomes 3 independent replicates

Fig. 7AD_raw_image

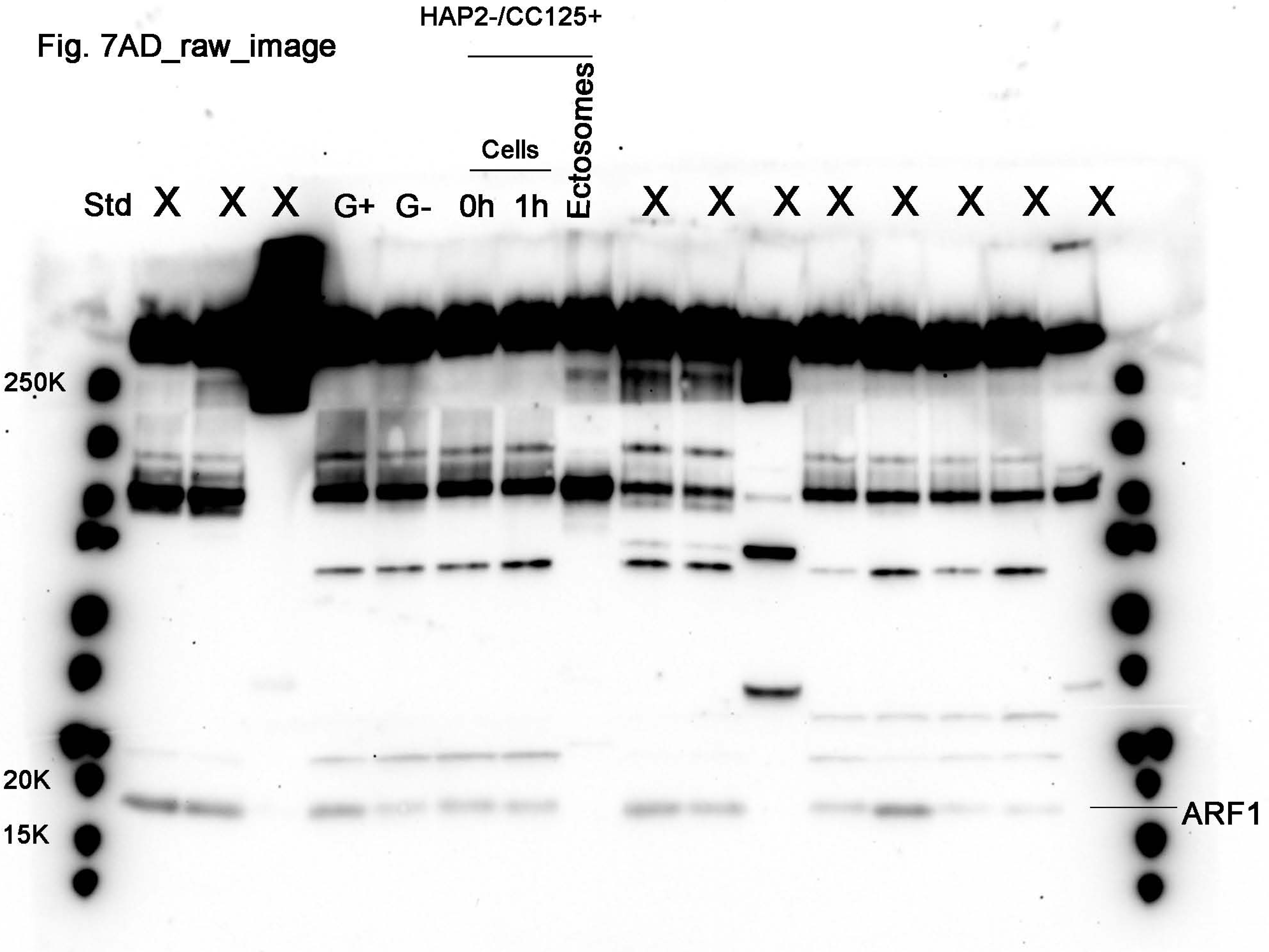

Fig. 7AD_coomassie stained

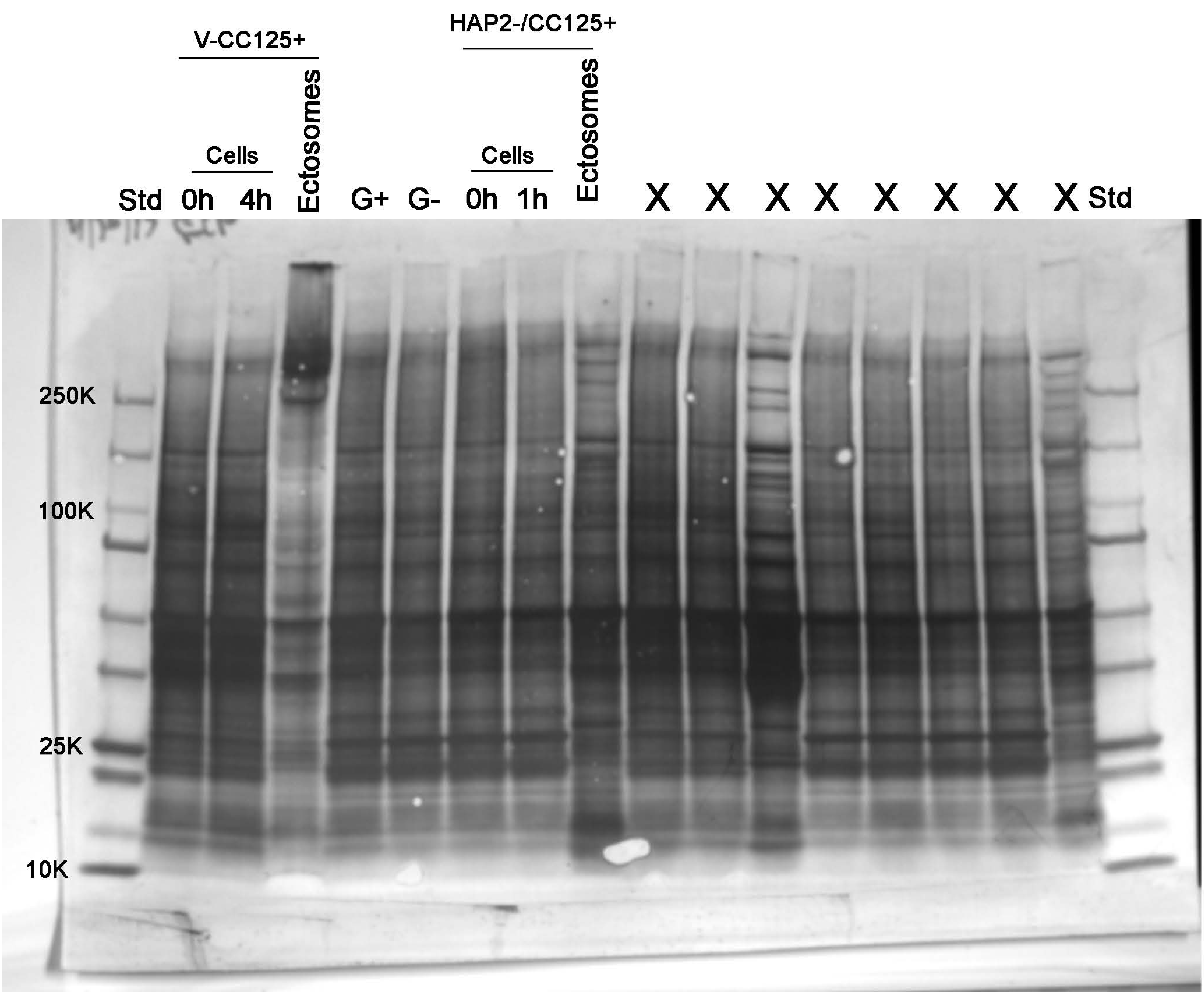

Fig. 7AD_raw_image

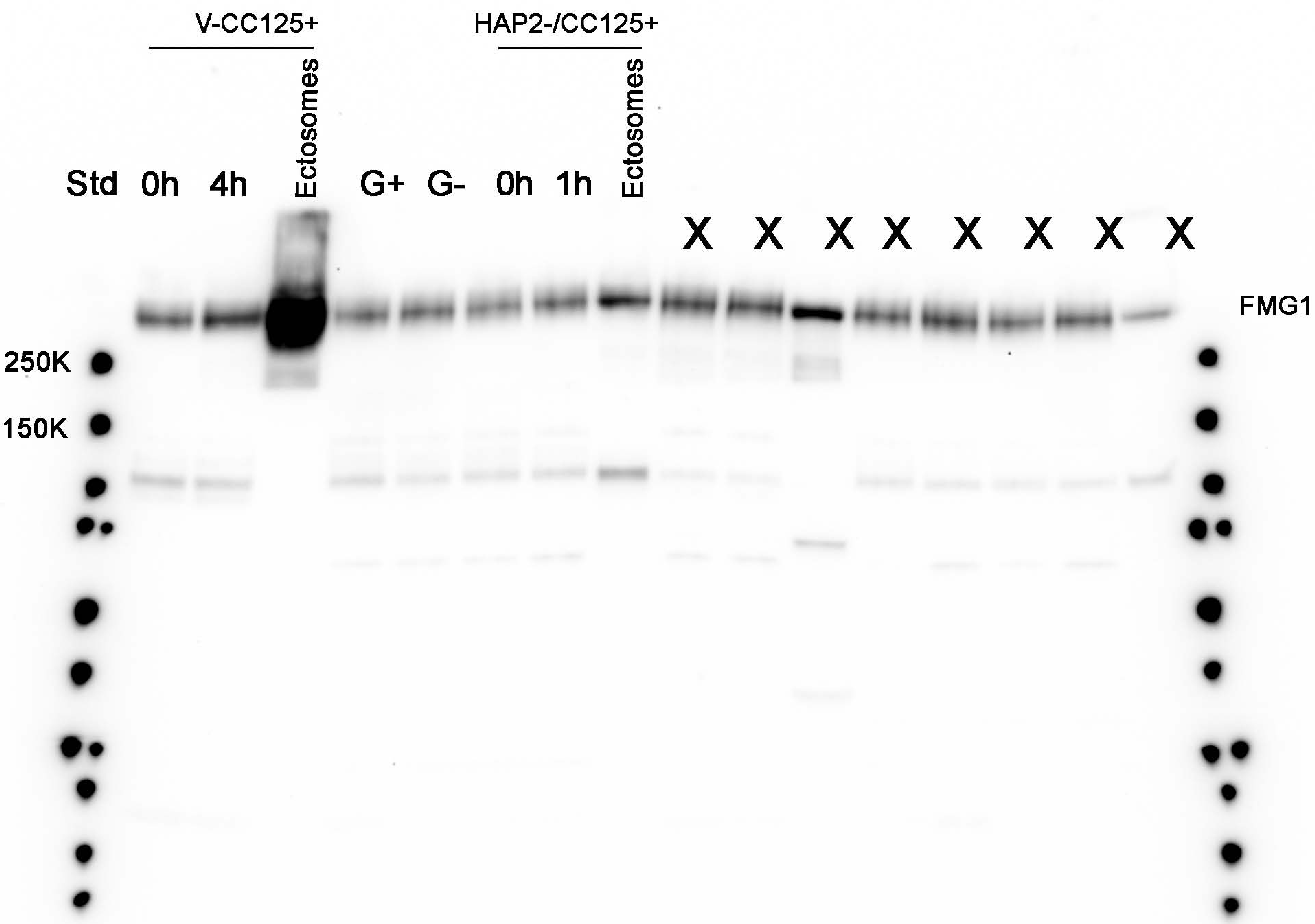

Fig. 7AD_raw_image

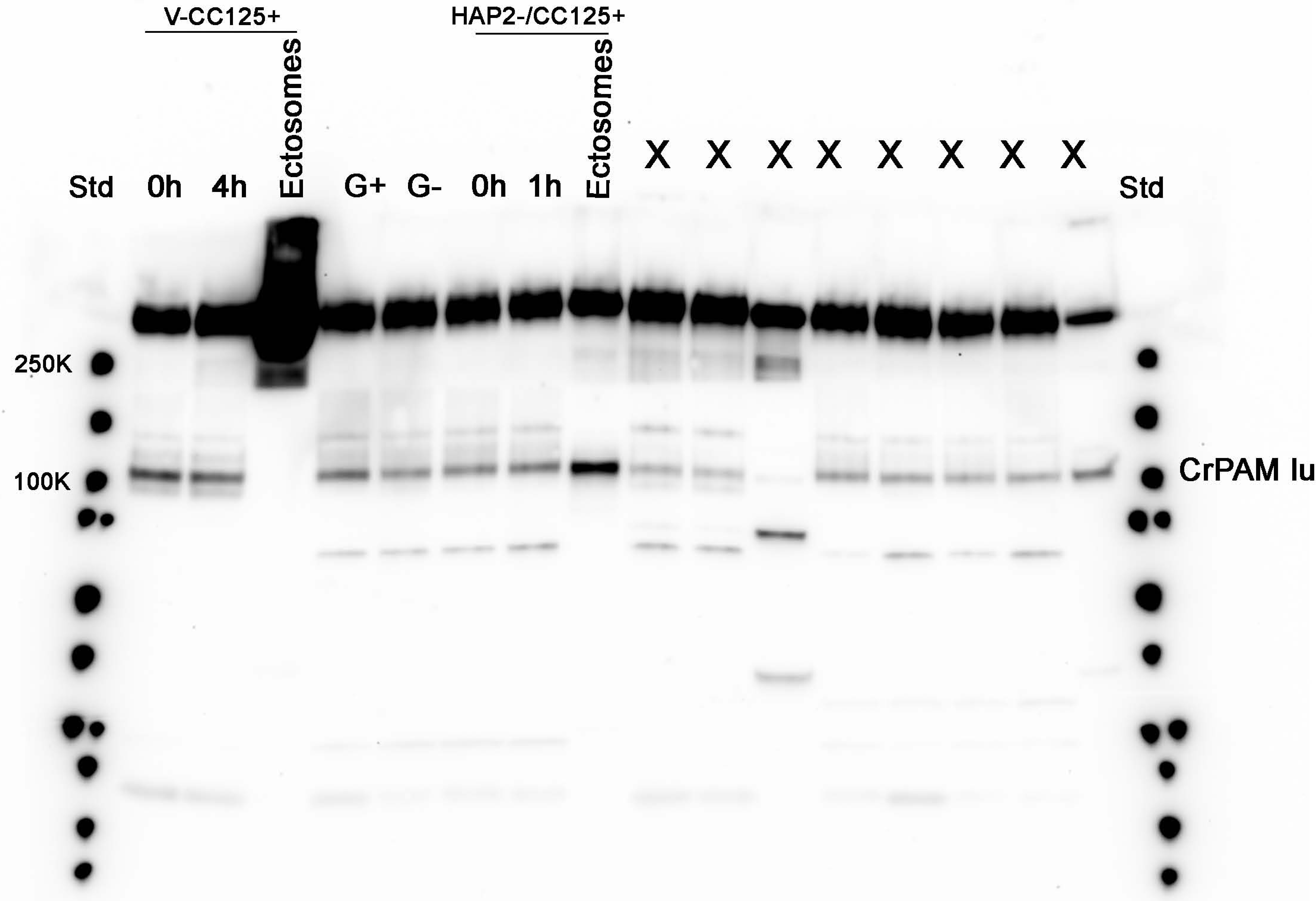

Fig. 7B_raw_image

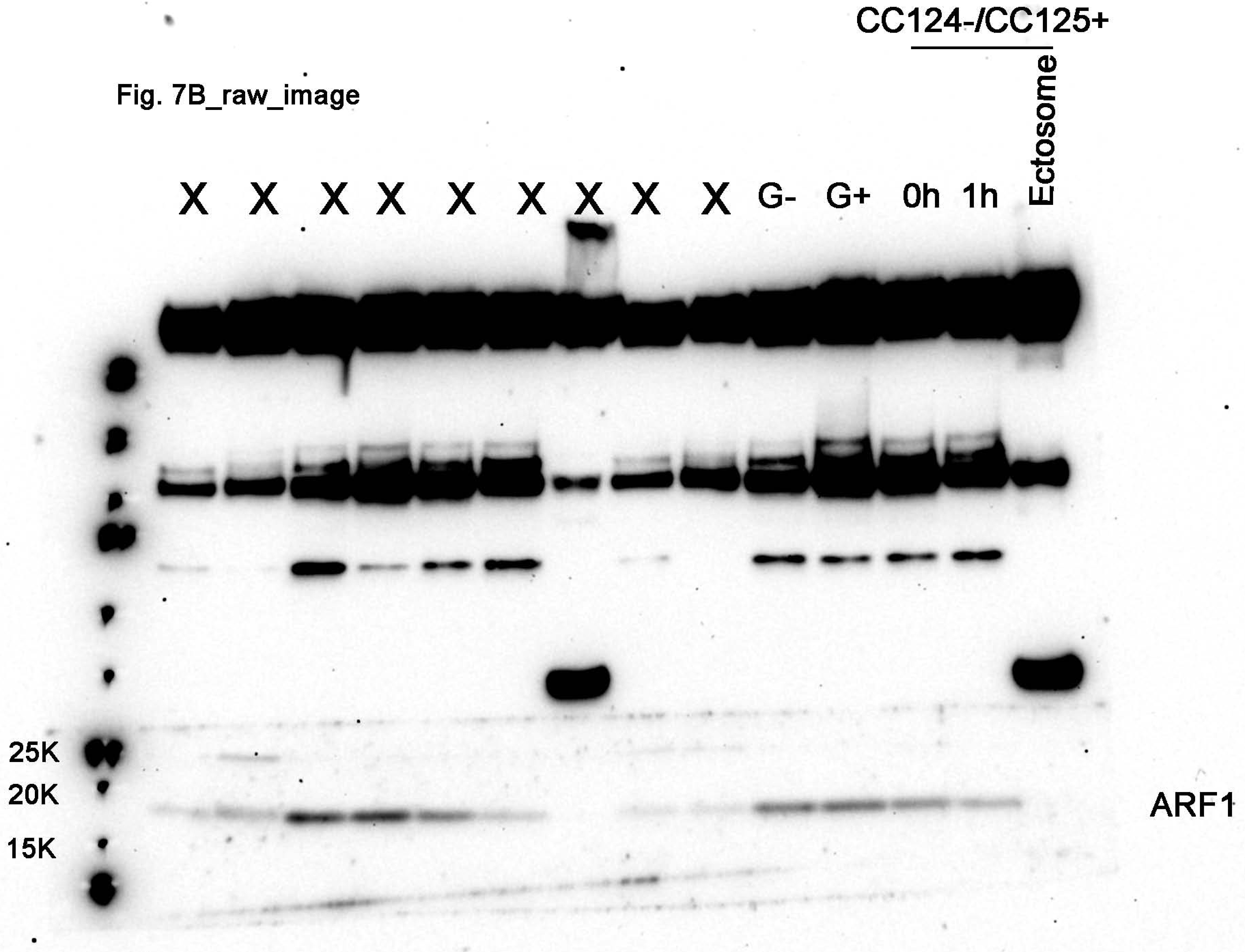

Fig. 7B_coomassie stained

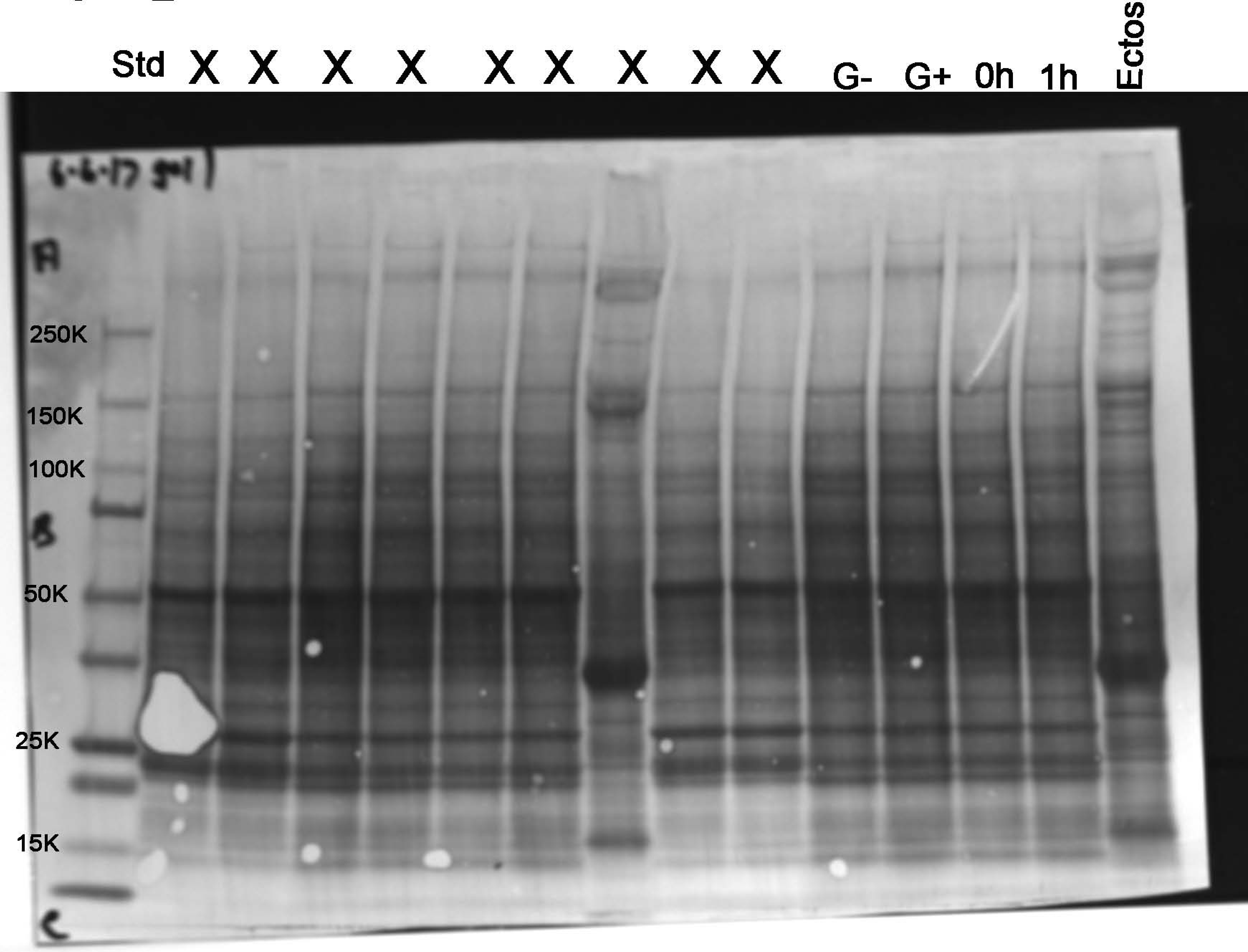

Fig. 7B_raw_image

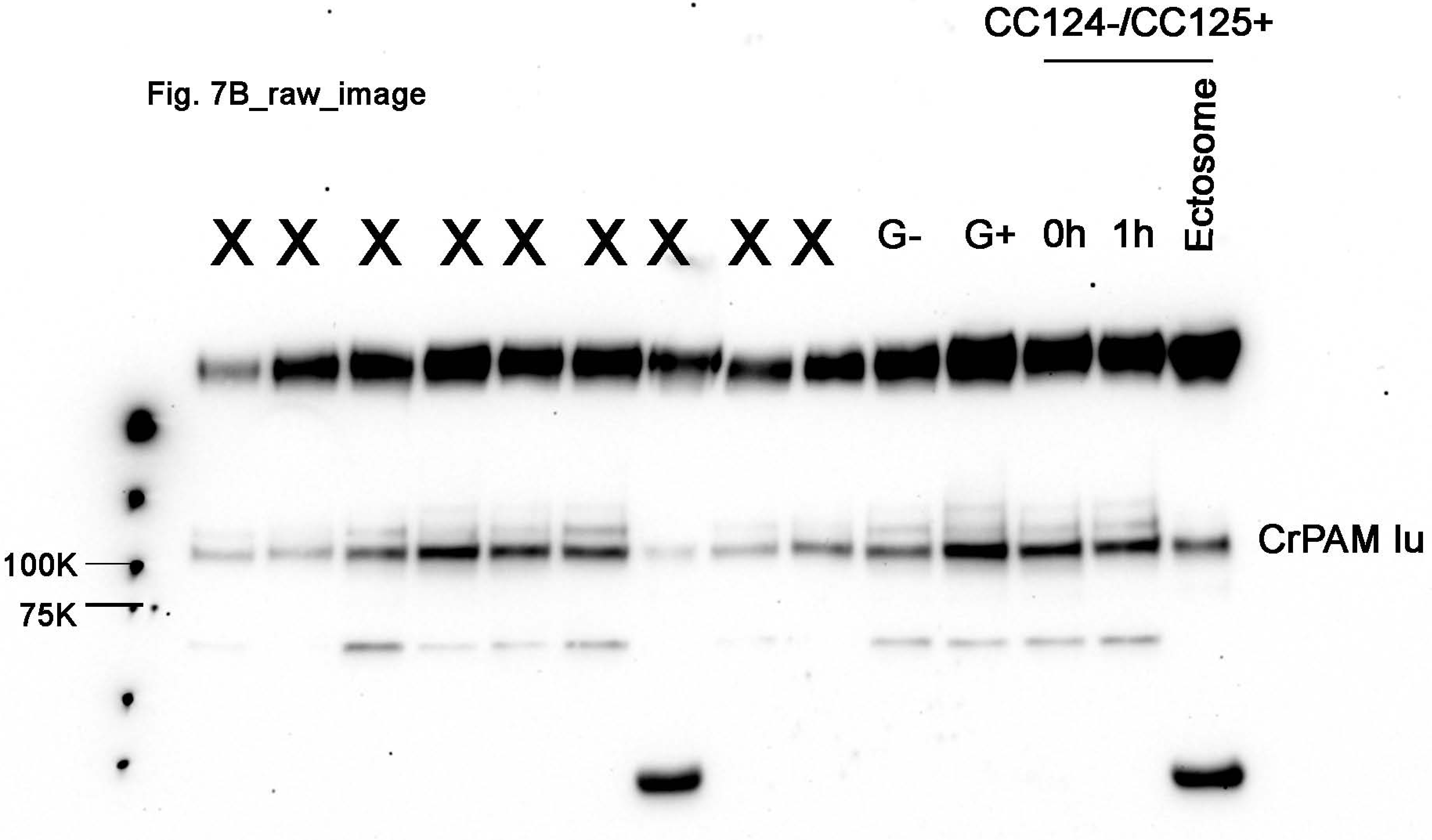

Fig. 7B_raw_image

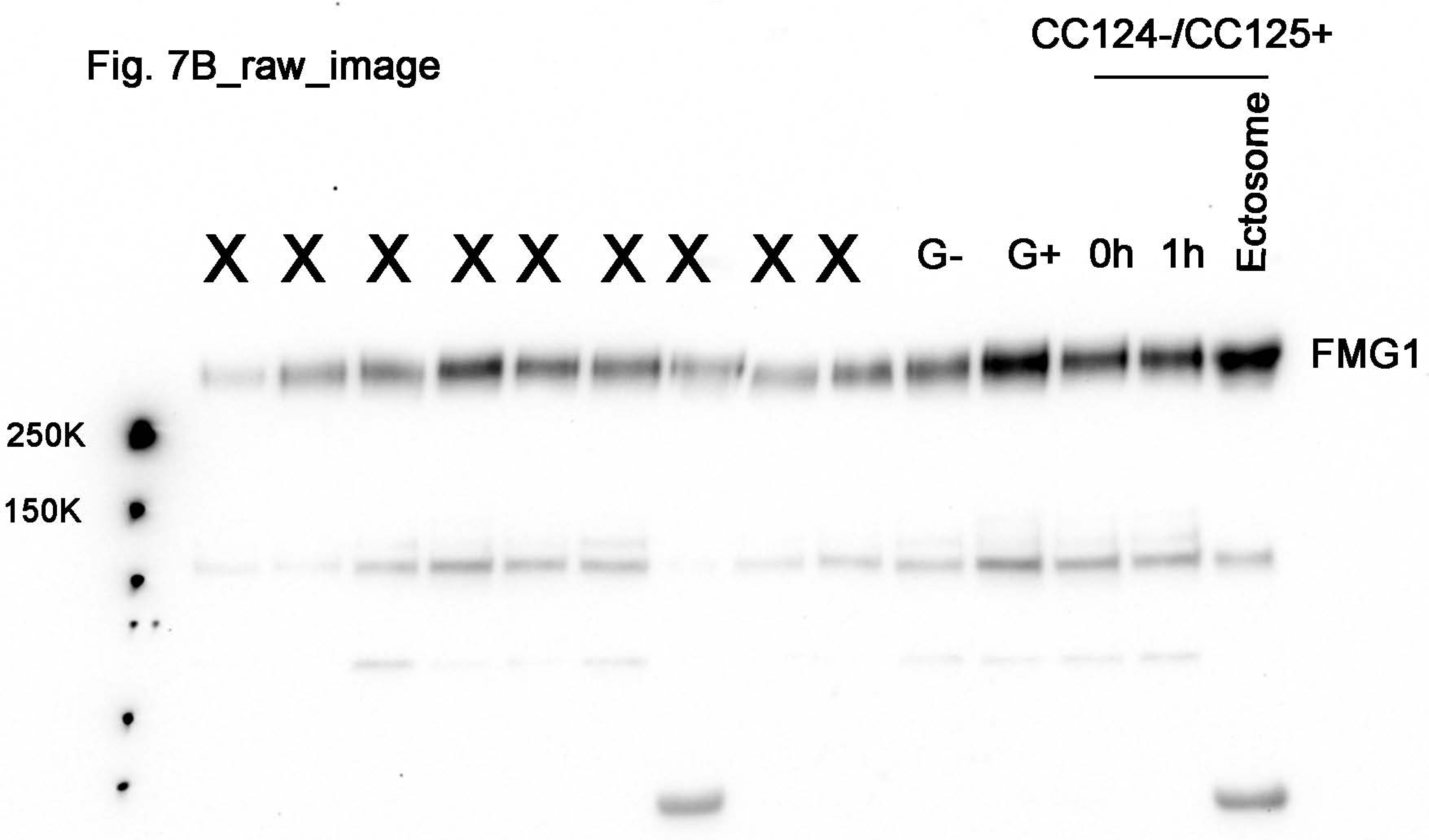

Figure 7C_raw_image

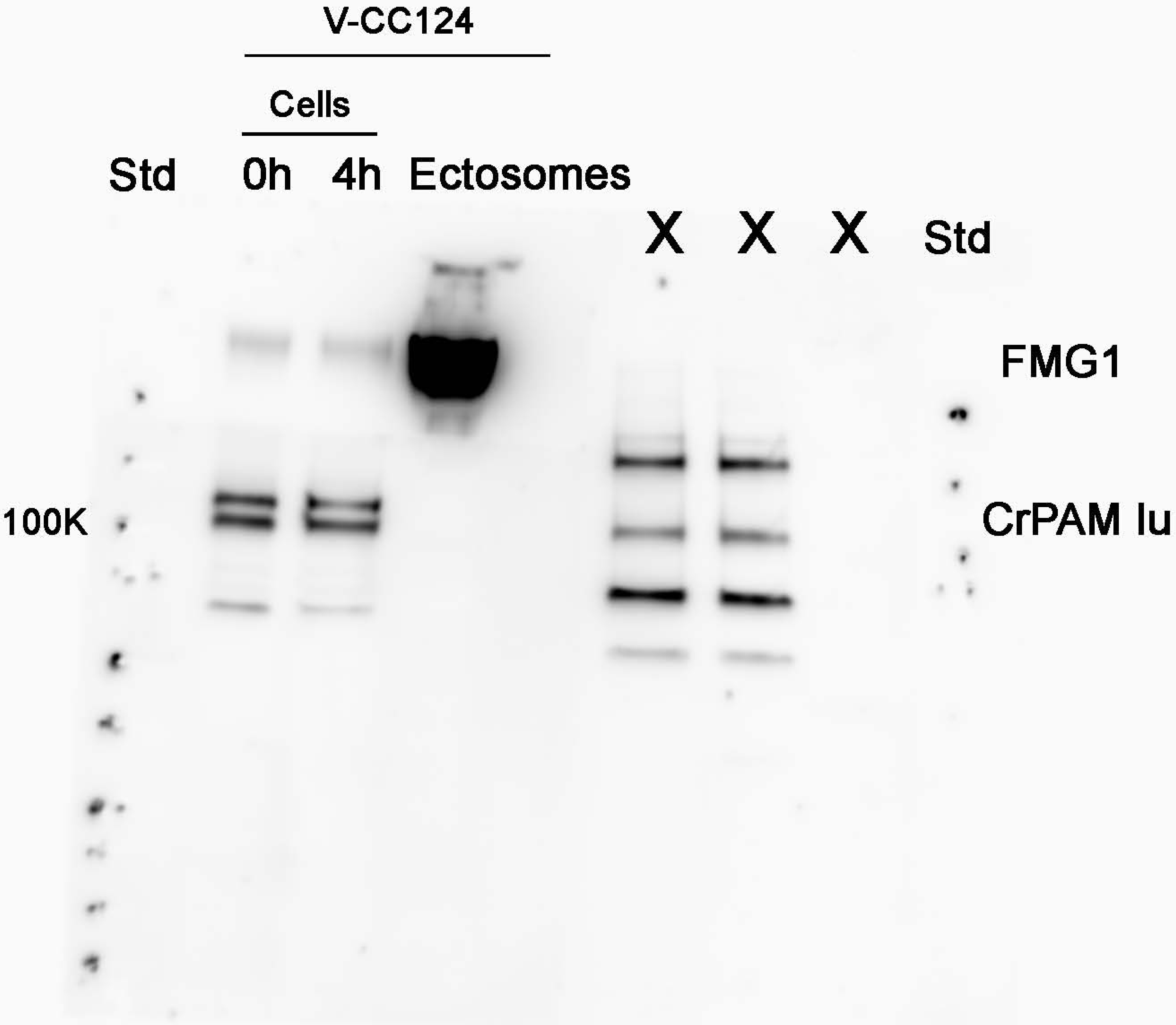

Figure 7C_coomassie staiened_raw_image

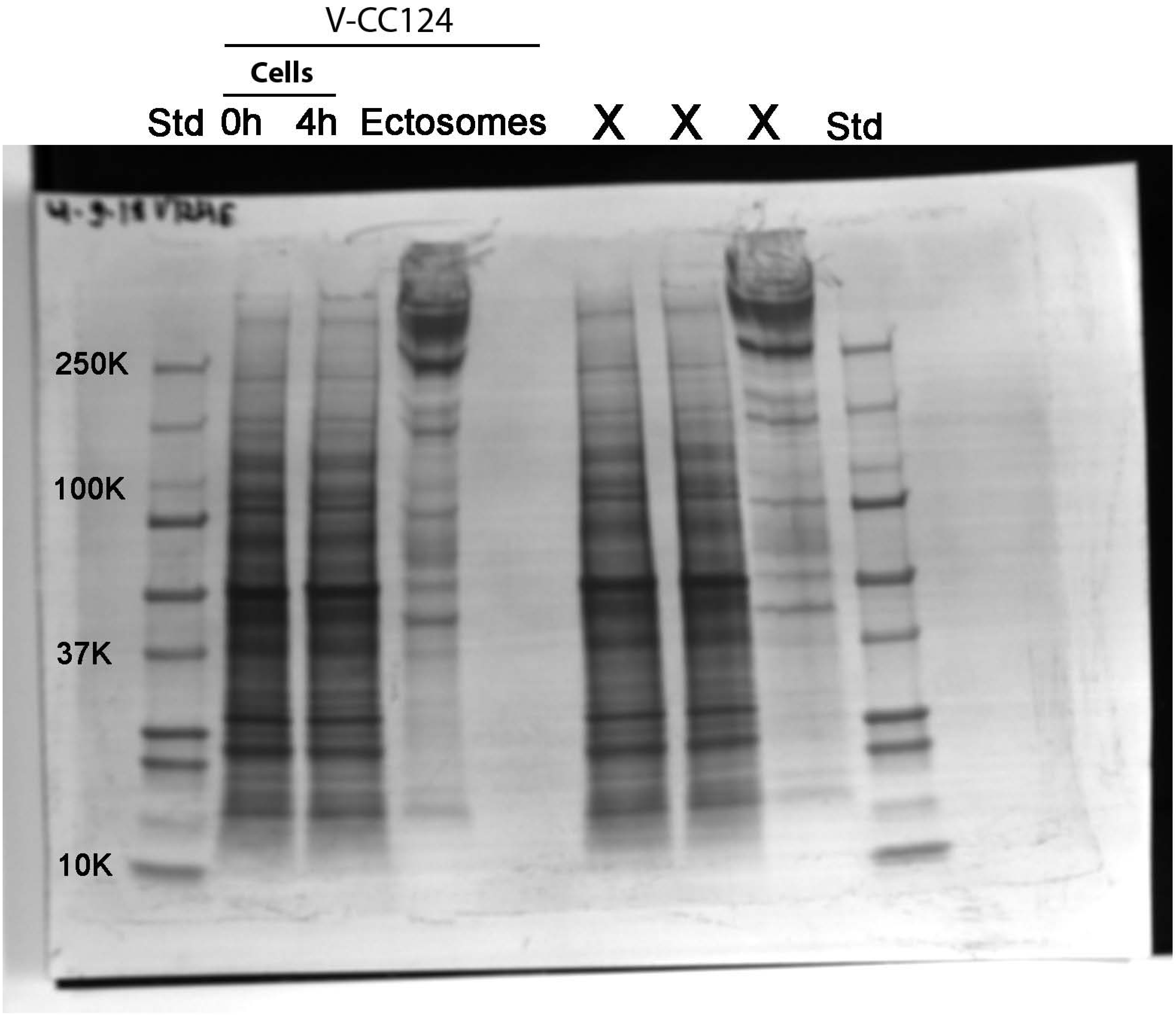

Fig. 8C_raw_image

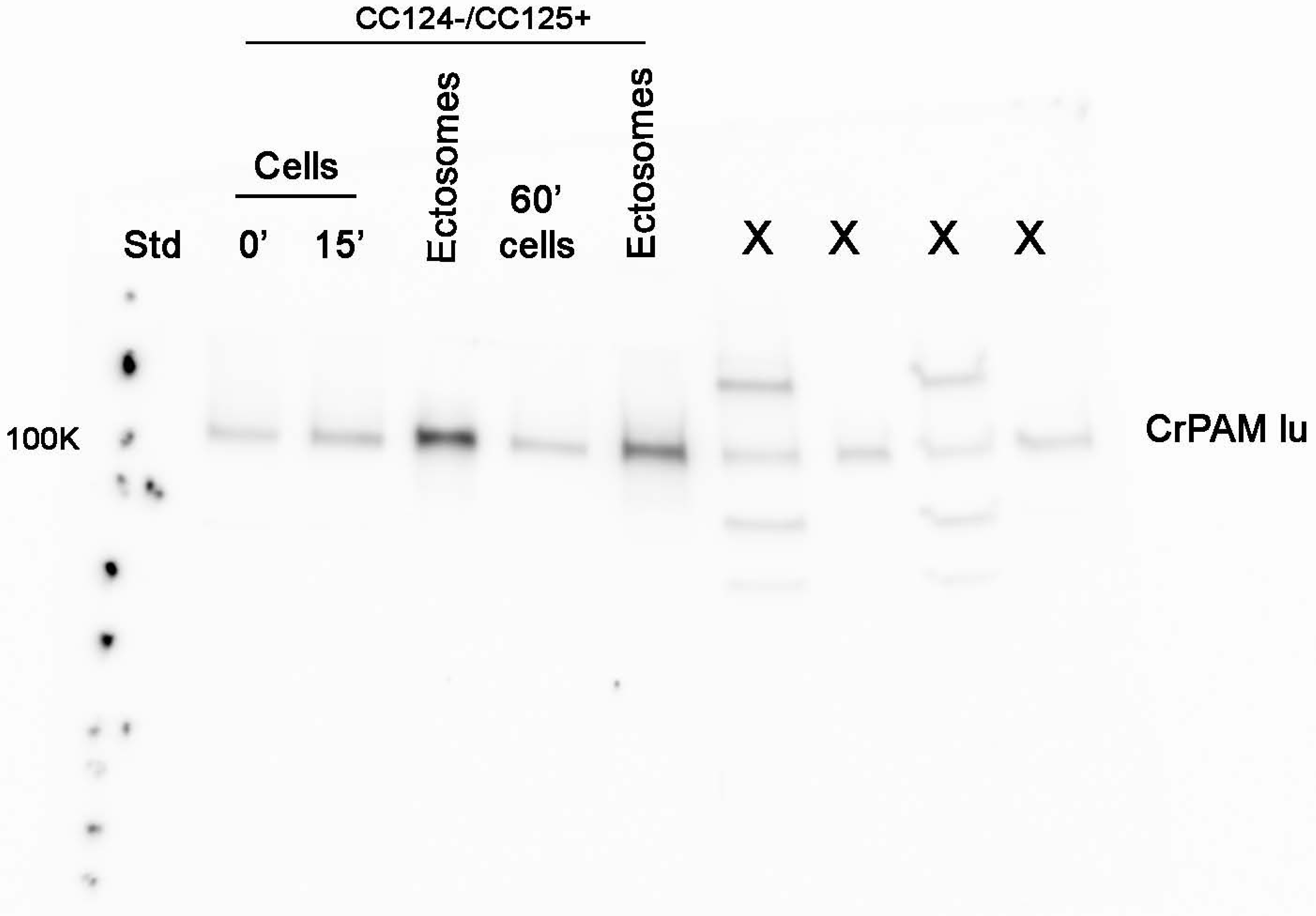

Fig. 8C_coomassie_raw_image

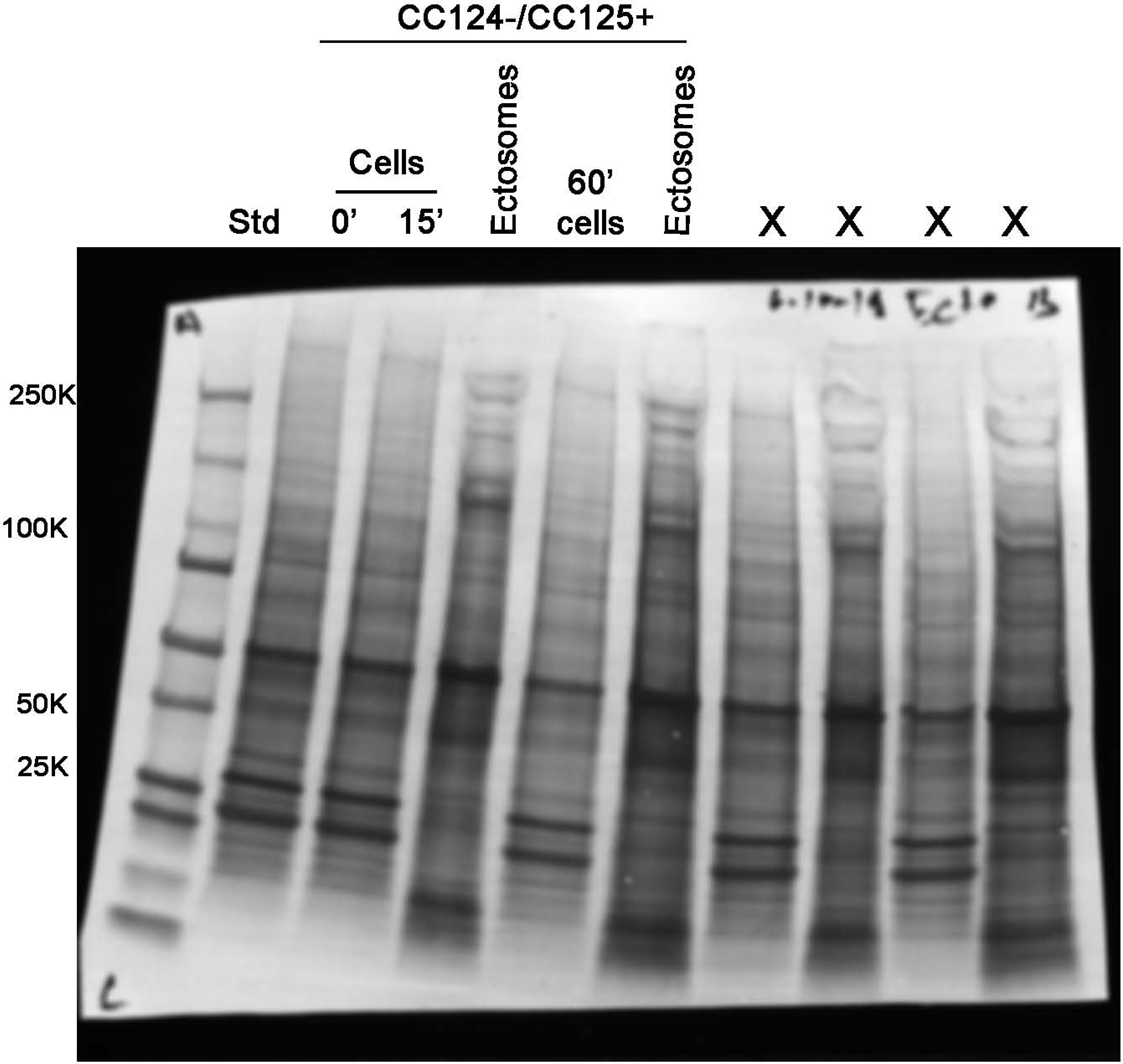

Supplement: S1 Raw Images — (PDF) [file pbio.3000566.s015.pdf]
